# Joint Models of Two Longitudinal Biomarkers and Clustered Survival Data for Cluster‐Informed Dynamic Prediction With Application to Periodontitis

**DOI:** 10.1002/sim.70715

**Published:** 2026-09-08

**Authors:** Sean Xinyang Feng, Laurent Briollais, Aya A. Mitani

**Affiliations:** ^1^ Division of Biostatistics, Dalla Lana School of Public Health University of Toronto Toronto Canada; ^2^ Lunenfeld‐Tanenbaum Research Institute, Mount Sinai Hospital Toronto Canada

**Keywords:** calibration, discrimination, dynamic prediction, joint model, multilevel data, periodontitis

## Abstract

Joint modeling of longitudinal data and time‐to‐event data have been extended to accommodate multilevel data structures. In dental studies, data often exhibit a multilevel hierarchy: each patient has multiple teeth, and one or more biomarkers are measured repeatedly over time for each tooth. In addition to biomarker measurements, the time to tooth loss may vary differently between patients as some patients are more susceptible to tooth loss, conditional on other risk factors. In this paper, we account for intra‐patient and intra‐tooth correlations in the longitudinal measurement of a continuous biomarker, probing pocket depth (PPD), and a binary biomarker, mobility. We also account for the correlation in time to tooth loss between teeth within the same patient. We jointly model the two longitudinal measurements and the risk of tooth loss using Bayesian estimation. We develop a cluster‐informed dynamic prediction framework for the survival outcome, in which the prediction for a given unit is informed not only by its own observed history but also by the observed longitudinal trajectories and event outcomes of other units within the same cluster. We evaluate the predictive performance in terms of discrimination and calibration, accounting for censoring and the multilevel data structure. Our simulation study shows that the proposed joint model produced more accurate estimates and better predictive performance compared to the standard bivariate joint model that ignores the multilevel data structure. We applied our model to electronic periodontal data obtained from the Canadian Armed Forces (CAF).

## 1 Introduction

In clinical studies, longitudinal data often come in the form of biomarkers that are repeatedly measured in patients over time, capturing the progression of the disease. Time‐to‐event data may refer to the time from the beginning of the study until the occurrence of an event of interest, such as death. Understanding how biomarkers affect the risk of an event is important when making clinical decisions. Joint modeling of longitudinal and time‐to‐event data have become a powerful tool to analyze and predict event risk using time‐varying biomarkers [1]. Rizopoulos [2] provided a comprehensive review of joint models and outlined various extensions that include discrete longitudinal biomarkers, multiple biomarkers, and advanced association structures. Other studies have expanded joint models to accommodate recurrent events [3, 4] and competing risks [5, 6, 7]. In addition, joint models can be used to dynamically predict survival probabilities, updating the risk based on biomarker trajectories [8, 9, 10, 11].

**FIGURE 1:**
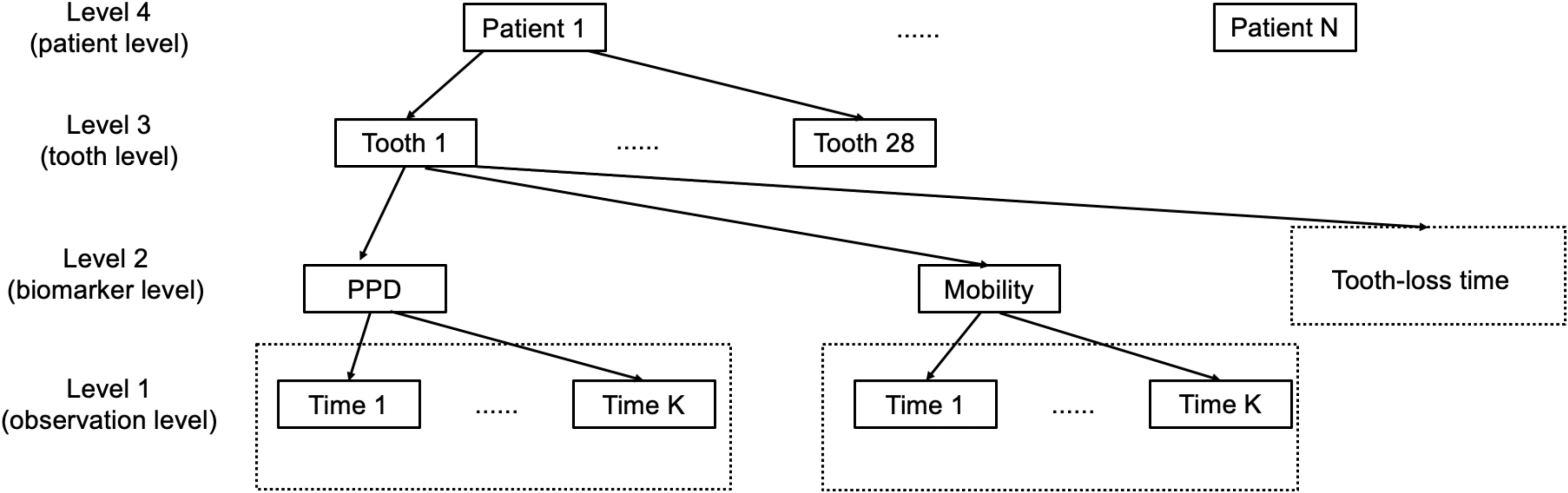
Multilevel data structure of dental data: patients (level 4) have multiple teeth (level 3), where two biomarkers PPD and mobility (level 2) are measured repeatedly over time (level 1).

The motivation of this paper comes from building a joint model and making dynamic predictions for tooth loss in patients with periodontitis, a prevalent, and serious infection of the gums and bones surrounding the teeth [12]. Severe periodontitis can destroy the support of periodontal tissue and cause teeth to loosen or even loss of teeth if left untreated [13]. Predicting the risk of tooth loss can guide timely interventions, help prioritize treatment for teeth most at risk, improve long‐term oral health outcomes, and support shared decision‐making between dentists and patients [14]. Diagnosis of periodontitis relies on periodontal biomarkers such as probing pocket depth (PPD) and tooth mobility. PPD measures the distance from the gingival margin to the bottom of the periodontal pocket in millimeters, and mobility indicates whether a tooth is loose. PPD and mobility are routinely measured at regular clinical visits to monitor disease progression. As illustrated in Figure 1, dental data exhibit a multilevel structure: patients (level 4) have multiple teeth (level 3), where biomarkers (level 2) are measured repeatedly over time (level 1). This structure introduces multiple levels of intra‐patient and intra‐tooth correlations that should be accounted for in longitudinal and survival models to avoid biased inferences [15]. Specifically, for each patient, the teeth are naturally clustered and correlated [16]. For each tooth, two biomarkers could be correlated at the same time and the longitudinal measurements of a given biomarker exhibit temporal correlation. Additionally, the time‐to‐event data of teeth within the same patient is also clustered.

Recently, joint models for clustered data have attracted increasing interest. Brilleman et al. [17] developed a joint model for multilevel data, incorporating nested random effects in the longitudinal submodel. However, their model assumed that the survival processes are independent of each other, and they lacked a simulation study. Other approaches assumed that the variation in survival processes of units within the same cluster is entirely captured through shared random effects in the longitudinal submodel. Kürüm et al. [18] proposed a joint model to study the effect of hospitalization on mortality in patients with end‐stage kidney disease nested within dialysis facilities. Mitani et al. [15] illustrated the use of joint models in estimating the effects of risk factors for tooth loss, accounting for within‐patient correlation using a group bootstrap algorithm. Lu et al. [19] developed a clustered trivariate joint model for longitudinal count data, recurrent events, and a terminal event for family data, using individual‐level and family‐level random intercepts to account for dependence among individuals and families. Their approach considered longitudinal count data instead of continuous data. Additionally, there remains a gap in accounting for more than one biomarker associated with clustered survival outcomes.

Dynamic prediction for clustered survival data has been relatively understudied. Rondeau et al. [20] investigated dynamic prediction for clustered survival times using frailty models. They proposed two types of predictive probabilities given the covariates: conditional predictive probabilities, which account for a specific cluster from the sample data, and marginal predictive probabilities, which are averaged over the population. Conditional prediction has less clinical interest because it is used for units of a specific cluster in the sample data. The marginal prediction focuses on the population and is preferred when the interest is on predicting events for units from a new cluster. Choi et al. [21] developed joint models for clustered recurrent and terminal events and made dynamic predictions of the terminal event in family studies. However, to our knowledge, no study has applied joint models to clustered data incorporating longitudinal biomarkers to perform dynamic predictions.

With respect to quantifying dynamic predictive accuracy, several methods have been proposed in non‐clustered settings. Mauguen et al. [22] introduced time‐varying Brier scores (BS) for frailty models and accounted for right‐censoring using nonparametric inverse probability of censoring weighting (IPCW). Blanche et al. [23] extended dynamic area under the ROC curve (AUC) and BS for joint models using nonparametric IPCW. Rizopoulos and Taylor [24] compared model‐based and IPCW‐based time‐varying BS in the joint modeling framework. However, these approaches do not address clustered data structures to evaluate time‐varying AUC.

Importantly, dynamic prediction for clustered data can differ conceptually from the standard dynamic prediction for non‐clustered data in the current literature. In non‐clustered settings, dynamic prediction for a subject is typically based on that subject's own longitudinal history [8, 9, 10, 11]. In clustered settings, however, survival outcomes for units within the same cluster are often correlated. Therefore, it motivates an extension of the definition of dynamic prediction, in which the prediction for a given unit is informed not only by its own observed history (unit‐informed) but also by the observed longitudinal trajectories and event outcomes of other units within the same cluster (cluster‐informed). This type of cluster‐informed dynamic prediction has not been formally developed in the existing literature.

In this paper, we aim to develop a joint model that addresses the multilevel clustering of periodontal data, allowing for within‐cluster correlation in both longitudinal and survival outcomes. In the longitudinal submodel, we introduce biomarker‐specific random effects at the patient‐level and tooth‐level with appropriate covariance structure. In the survival submodel, we introduce a shared frailty term at the tooth‐level to capture correlation in the survival times between teeth within the same patient. Based on the proposed joint model, we derive dynamic predictions for the survival outcome of a given unit conditional on the longitudinal information and survival outcome of all units within the same cluster. We further evaluate the predictive performance in terms of discrimination and calibration while accounting for censoring and clustering. Our primary interest is in marginal prediction, which is most relevant for predicting the time to tooth loss for a new cluster from the same population.

The remainder of this article is organized as follows. In Section 2, we present the proposed joint model of two longitudinal biomarkers and clustered survival data. We develop cluster‐informed dynamic prediction using the proposed joint model and evaluate the predictive performance. Section 3 presents simulation studies that assess the performance of the proposed methods. In Section 4, we apply our model to an electronic periodontal data set. Section 5 concludes with a discussion and future research directions.

## 2 Method

We consider clustered longitudinal data with repeated biomarker measurements and terminal events. Let i=1,…,N denote the index of independent clusters (patient), $j=1,\ldots ,n_{i}$ denote the unit (tooth) of a cluster, where $n_{i}$ is the size (number of teeth) of cluster i, and $k=1,\ldots ,m_{ij}$ denote the observation index for each unit j in cluster i, with $m_{ij}$th visit corresponding to the last follow‐up or the final visit before the terminal event. Let $Y_{Aij}(t)=Y_{Aijk}$ and $Y_{Bij}(t)=Y_{Bijk}$ denote the measurements of a continuous and a binary longitudinal biomarker, respectively, observed at time t, corresponding to the kth observation. We denote cluster‐level and unit‐level covariates by $X_{i}(t)={\left\{X_{i1}(t),\ldots ,X_{ip}(t)\right\}}^{T}$ and $Z_{ij}(t)={\left\{Z_{ij1}(t),\ldots ,Z_{ijq}(t)\right\}}^{T}$, respectively. Note that $X_{i}(t)$ and $Z_{ij}(t)$ are possibly time‐dependent. We observe an event time $T_{ij}=\min\{T_{ij}^{*},C_{ij}\}$, where $T_{ij}^{*}$ denotes the true event time of unit ij, $C_{ij}$ is the right‐censoring time. We let $\delta_{ij}$ be the event indicator, where $\delta_{ij}=1$ if $T_{ij}=T_{ij}^{*}$ and $\delta_{ij}=0$ if $T_{ij}=C_{ij}$.

### 2.1 Joint Model Definition

The joint model framework models the joint distribution of longitudinal and survival outcomes. To account for the multilevel data structure, we specify a mixed‐effects model with nested random effects for each longitudinal biomarker as follows:

(1) $$\begin{array}{ll} Y_{Aij}(t) & =\eta_{Aij}(t)+\epsilon_{ij}(t)=X_{i}^{T}(t)\beta_{AX}+Z_{ij}^{T}(t)\beta_{AZ}+b_{Ai}\\ & \;+b_{Aij,0}+b_{Aij,1}t+\epsilon_{ij}(t), \end{array}$$

(2) $$\begin{array}{ll} \text{logit}\{E[Y_{Bij}(t)]\} & =\eta_{Bij}(t)=X_{i}^{T}(t)\beta_{BX}+Z_{ij}^{T}(t)\beta_{BZ}\\ & \;+b_{Bi}+b_{Bij,0}+b_{Bij,1}t, \end{array}$$

where $\eta_{Aij}(t)$ and $\eta_{Bij}(t)$ are linear predictors for each biomarker, $\beta_{AX}$, and $\beta_{AZ}$ are the cluster‐level and unit‐level coefficients for biomarker $Y_{A}$, and $\beta_{BX}$ and $\beta_{BZ}$ are the respective coefficients for biomarker $Y_{B}$. Additionally, $\epsilon_{ij}(t)∼N(0,\sigma_{e}^{2})$. The biomarker‐specific cluster‐level random effects, $b_{i}={\left(b_{Ai},b_{Bi}\right)}^{T}$, captures the variability within clusters for both biomarkers. The biomarker‐specific unit‐level random effects, $b_{ij}={\left(b_{Aij},b_{Bij}\right)}^{T}={\left(b_{Aij,0},b_{Aij,1},b_{Bij,0},b_{Bij,1}\right)}^{T}$, captures the variability within units for both biomarkers over time. We assume that $b_{i}∼MVN(0,\sum_{C})$ and $b_{ij}∼MVN(0,\sum_{U})$. The covariance matrices $\sum_{C}$ and $\sum_{U}$ are assumed to be unstructured. They capture the correlations within and across biomarkers at different times and units within the same cluster. We let $\rho_{C}$ denote the correlation between cluster‐level random effects $b_{Ai}$ and $b_{Bi}$, and $\rho_{1},\ldots ,\rho_{6}$ represent the correlations among unit‐level random effects, ${\left(b_{Aij,0},b_{Aij,1},b_{Bij,0},b_{Bij,1}\right)}^{T}$. We assume independence between $b_{i}$ and $b_{ij}$.

Given the above parametrization, the marginal covariance between the linear predictors, $\eta_{Aij}(t)$, and $\eta_{Bij}(t)$, considering different times, units, and biomarkers, are given in Supporting Information A. These covariance structures capture the correlations within and between biomarkers at different times and units within the same cluster.

The hazard rate of the event for each unit ij is specified with a frailty model to account for the unobserved cluster‐level heterogeneity in addition to the longitudinal trajectories and the effects of $X_{i}(t)$ and $Z_{ij}(t)$:

(3) $$\begin{array}{ll} h_{ij}(t) & =h_{0}(t)\exp\{r_{i}+X_{i}^{T}(t)\gamma_{X}+Z_{ij}^{T}(t)\gamma_{Z}\\ & \;+f(\alpha_{1},\eta_{Aij}(t))+f(\alpha_{2},\eta_{Bij}(t))\}, \end{array}$$

where $h_{0}(t)$ is the baseline hazard rate, $r_{i}$ is the cluster‐level frailty term assumed to follow a normal distribution with $r_{i}∼N(0,\sigma_{r}^{2})$, $\gamma_{X}$ and $\gamma_{Z}$ are coefficient vectors for $X_{i}(t)$ and $Z_{ij}(t)$, respectively, $\alpha_{1}$ and $\alpha_{2}$ are the association parameters that quantify the effects of the biomarker processes, $\eta_{Aij}(t)$ and $\eta_{Bij}(t)$, at time t on the hazard of the event. The function f() is the association structure that specifies the dependence between the longitudinal and survival processes. Common association structures include the underlying value of the longitudinal biomarker at time t, both the value and the slope of the longitudinal biomarker at t, and the cumulative exposure to the biomarker trajectory up to time t.

### 2.2 Joint Model Estimation

We denote the vector of population‐level parameters by $\theta =(\theta_{y},\theta_{S})$, where $\theta_{y}$ is the vector of parameters from the longitudinal submodel, $\theta_{y}=(\beta_{AX},\beta_{BX},\beta_{AZ},\beta_{BZ},\sum_{C},\sum_{U},\sigma_{e})$, and $\theta_{S}$ is the vector of parameters from the survival submodel, $\theta_{S}=(\gamma_{X},\gamma_{Z},\alpha_{1},\alpha_{2},\sigma_{r})$. For convenience, we let $\beta =(\beta_{AX},\beta_{BX},\beta_{AZ},\beta_{BZ})$, $\gamma =(\gamma_{X},\gamma_{Z})$, and $\alpha =(\alpha_{1},\alpha_{2})$.

We assume that conditional on the random effects $\left(b_{i},b_{ij}\right)$ and the population‐level parameters θ, the longitudinal outcomes of unit ij at time t are independent to the survival outcome, each longitudinal biomarker measurements are independent between two time points, and independent from each other at the same time point. That is,

(4) $$\begin{array}{l} \{Y_{Aij}(t),Y_{Bij}(t)\}⟂\{T_{ij},\delta_{ij}\}\;|\;b_{i},b_{ij},\theta \end{array}$$

(5) $$\begin{array}{l} Y_{Aij}(t)⟂Y_{Aij}(t^{\prime })\;|\;b_{i},b_{ij},\theta \end{array}$$

(6) $$\begin{array}{l} Y_{Bij}(t)⟂Y_{Bij}(t^{\prime })\;|\;b_{i},b_{ij},\theta \end{array}$$

(7) $$\begin{array}{l} Y_{Aij}(t)⟂Y_{Bij}(t)\;|\;b_{i},b_{ij},\theta \end{array}$$

Additionally, conditional on the cluster‐level random effect $b_{i}$, the frailty term $r_{i}$ and the population‐level parameters θ, the survival outcomes of units within the same cluster are assumed to be independent. That is, $\{T_{ij},\delta_{ij}\}⟂\{T_{ij^{\prime }},\delta_{ij^{\prime }}\}\;|\;b_{i},r_{i},\theta$.

Under these conditional independence assumptions, the log‐posterior distribution contributed from cluster i is given by:

$$\begin{array}{ll} & \log\;p(b_{i},b_{ij},r_{i};\theta |Y_{i},T_{i},\delta_{i})\\ & \;=\overset{n_{i}}{\underset{j=1}{\sum }}\overset{m_{ij}}{\underset{k=1}{\sum }}\log\;p(Y_{Aijk}|\;b_{i},b_{ij},\theta )+\overset{n_{i}}{\underset{j=1}{\sum }}\overset{m_{ij}}{\underset{k=1}{\sum }}\log\;p(Y_{Bijk}|\;b_{i},b_{ij},\theta )\\ & \;+\overset{n_{i}}{\underset{j=1}{\sum }}\log\;p(T_{ij},\delta_{ij}|\;b_{i},b_{ij},r_{i},\theta )+\overset{n_{i}}{\underset{j=1}{\sum }}\log\;p(b_{ij}|\theta )+\log\;p(b_{i}|\theta )\\ & \;+\log\;p(r_{i}|\theta )+\log\;p(\theta )+C \end{array}$$

where $\log\;p(Y_{Aijk}|\;b_{i},b_{ij},\theta )$ and $\log\;p(Y_{Bijk}|\;b_{i},b_{ij},\theta )$ are the log‐likelihood functions for longitudinal submodels (1) and (2), respectively, $\log\;p(T_{ij},\delta_{ij}|\;b_{i},b_{ij},r_{i},\theta )$ is the log‐likelihood function for the survival submodel (3), $\log\;p(b_{i}|\theta )$ and $log\;p(b_{ij}|\theta )$ represent the log‐likelihoods for the distribution of cluster‐level and unit‐level random effects, logp(θ) is the log‐likelihood for the joint prior distribution of all parameters, and C is a constant.

To make inference of the parameters, we may use likelihood‐based methods such as the expectation‐maximization algorithm [25]. However, such approach requires integrating out the distribution of random effects, which is computationally expensive and analytically intractable due to the high dimension of random effects [26]. Therefore, we proceed with Bayesian sampling methods to make inference. We estimate the model parameters using Hamiltonian Monte Carlo implemented in Stan [27]. The prior distributions for the parameters are specified as follows: the regression parameters (β,γ,α) are assigned normal priors, the standard deviation of the residual errors, $\sigma_{e}$, is assigned a half‐Cauchy prior, the covariance matrix $\sum_{C}$ and $\sum_{U}$ are decomposed into correlation matrices and standard deviations. Each correlation matrix is specified using an LKJ prior, and each standard deviation is assigned a half Cauchy prior [28].

### 2.3 Cluster‐Informed Dynamic Prediction

Joint models provide a valuable tool for making dynamic predictions, allowing the estimation of individualized survival probabilities as new longitudinal information becomes available. For clustered data, it is often clinically important to predict the survival outcome of a given unit using not only its own longitudinal history but also the longitudinal trajectories and event information from other units within the same cluster.

To predict the survival probability for a new target unit $j^{*}$ in a new cluster i from the same population given that $j^{*}$ has survived up to time t (i.e., $T_{ij^{*}}^{*}>t$), we introduce the following notation:

- $Y_{ij^{*}}(t)=\{Y_{Aij^{*}}(\omega ),Y_{Bij^{*}}(\omega );\;0\leq \omega \leq t\}$ is the longitudinal biomarker measurements of the target unit $ij^{*}$ up to time t.
- ${𝒟}_{i-j^{*}}(t)=\{T_{ij}(t),\delta_{ij}(t),Y_{ij}(t);\;\text{for}\;j=1,\ldots ,n_{i}\;\text{and}\;j\neq j^{*}\}$ denotes the observed history of longitudinal biomarker measurements and survival outcomes from all units in cluster i excluding $j^{*}$ up to time t. The observed event time until t is defined as $T_{ij}(t)=\min\{T_{ij}^{*},C_{ij},t\}$, where $T_{ij}^{*}$ denotes the true event time of unit ij and $C_{ij}$ is the right‐censoring time. The event indicator $\delta_{ij}(t)=1$ if $T_{ij}(t)=T_{ij}^{*}$ and $\delta_{ij}(t)=0$ otherwise, for $j=1,\ldots ,n_{i}$ and $j\neq j^{*}$.
- $𝒟=\{T_{ij},\delta_{ij},Y_{Aijk},Y_{Bijk},X_{i}(t),Z_{ij}(t);\;\text{for}\;i=1,\ldots ,N,\;j=1,\ldots ,n_{i},\;k=1,\ldots ,m_{ij}\}$ denotes the sample data used to fit the joint model.

We assume dependence on cluster‐level and unit‐level covariates but omit the notation explicitly for ease of exposition. The dynamic prediction of survival probability of the target unit $ij^{*}$ can be formulated as the conditional survival probability of surviving beyond time u>t, given survival up to time t, the target unit's biomarker history $Y_{ij^{*}}(t)$, the observed information from the other units in the same cluster ${𝒟}_{i-j^{*}}(t)$, and the sample data 𝒟. Therefore, the cluster‐informed unit‐level dynamic prediction is defined as:

$$\pi_{ij^{*}}(u|t)=\mathrm{Pr}(T_{ij^{*}}^{*}\geq u|T_{ij^{*}}^{*}>t,Y_{ij^{*}}(t),{𝒟}_{i-j^{*}}(t),𝒟).$$

Let $B_{i}={\left[b_{i},\{b_{ij};\;j=1,\ldots ,n_{i}\},r_{i}\right]}^{T}$ denote the vector of cluster‐level random effects, all unit‐level random effects, and frailty term of cluster i. We assume that conditional on the parameters θ, the data from the new cluster and the sample data are independent, formally, $\{T_{ij^{*}},Y_{ij^{*}}(t),{𝒟}_{i-j^{*}}(t)\}\;⟂\;𝒟\;|\;\theta$. Under the conditional independence assumptions as shown in Equations (4) to (7), $\pi_{ij}(u|t)$ can be rewritten as

(8) $$\begin{array}{ll} \pi_{ij^{*}}(u|t) & =\int \frac{S_{ij^{*}}\{u|{ℋ}_{ij^{*}}(u,b_{i},b_{ij^{*}},r_{i},\theta );\theta \}}{S_{ij^{*}}\{t|{ℋ}_{ij^{*}}(t,b_{i},b_{ij^{*}},r_{i},\theta );\theta \}}\\ & \;p\{B_{i}|T_{ij^{*}}^{*}>t,Y_{ij^{*}}(t),{𝒟}_{i-j^{*}}(t);\theta \}p(\theta |𝒟)d(B_{i},\theta ), \end{array}$$

where

$$\begin{array}{ll} S_{ij^{*}}\{t|{ℋ}_{ij^{*}}(t,b_{i},b_{ij^{*}},r_{i},\theta );\theta \} & =\;\exp\left\{-\int_{0}^{t}h_{0}(\omega )\exp[r_{i}\right.\\ & \;+X_{i}^{T}(\omega )\gamma_{X}+Z_{ij^{*}}^{T}(\omega )\gamma_{Z}\\ & \;\left.+\alpha_{1}\eta_{Aij^{*}}(\omega )+\alpha_{2}\eta_{Bij^{*}}(\omega )]d\omega \right\} \end{array}$$

is the survival function of unit $ij^{*}$, and ${ℋ}_{ij^{*}}(\;)$ is the longitudinal history approximated by the linear mixed effect models. Since the integral of the survival function does not have a closed‐form solution, we use the Gaussian‐Kronrod quadrature method [29] for numerical approximation. The derivation of Equation (8) is provided in Supporting Information B.

In Equation (8), the second term of the integrand, $p\{B_{i}|T_{ij^{*}}^{*}>t,Y_{ij^{*}}(t),{𝒟}_{i-j^{*}}(t);\theta \}$, is the posterior distribution of the random effects of cluster i given its observed history from all units and the parameter vector θ. The third term, p(θ|𝒟), is the posterior distribution of the joint model parameters based on the sample data. We rewrite the log‐posterior distribution of the random effects as

(9) $$\begin{array}{cc} & \log\;p\{B_{i}|T_{ij^{*}}^{*}>t,Y_{ij^{*}}(t),{𝒟}_{i-j^{*}}(t);\theta \}\\ & \;\propto \log\;p\{T_{ij^{*}}^{*}>t,Y_{ij^{*}}(t),{𝒟}_{i-j^{*}}(t)|B_{i};\theta \}+\log\;p(B_{i};\theta )\\ & \;=\log\;p\{T_{ij^{*}}^{*}>t|b_{i},b_{ij^{*}},r_{i};\theta \}\\ & \;+\overset{n_{i}}{\underset{j=1,j\neq j^{*}}{\sum }}\log\;p\{T_{ij}(t),\delta_{ij}|\;b_{i},b_{ij},r_{i},\theta \}\\ & \;+\overset{n_{i}}{\underset{j=1}{\sum }}\log\;p\{Y_{ij}(t)|b_{i},b_{ij},r_{i};\theta \}+\log\;p(B_{i};\theta ). \end{array}$$

We estimate $\pi_{ij^{*}}(u|t)$ using an Monte Carlo procedure. Let $L_{m}$ denote the number of posterior draws of the model parameters. For each $l_{m}=1,\ldots ,L_{m}$, we perform the following steps:

1. Draw model parameters $\theta^{\left(l_{m}\right)}$ from the posterior distribution p(θ|𝒟) using MCMC samples.
2. Conditional on $\theta^{\left(l_{m}\right)}$, draw random effects $B_{i}^{\left(l_{m},1\right)},\ldots ,B_{i}^{\left(l_{m},L_{h}\right)}$, from the posterior distribution $p\{B_{i}|T_{ij^{*}}^{*}>t,Y_{ij^{*}}(t),{𝒟}_{i-j^{*}}(t);\theta^{\left(l_{m}\right)}\}$. The corresponding log‐posterior distribution is given in Equation (9). Because this conditional distribution has no closed‐form, we obtain $L_{h}$ samples using Hamiltonian Monte Carlo implemented in Stan.
3. For the target unit $ij^{*}$, compute
$$\begin{array}{ll} \pi_{ij^{*}}^{\left(l_{m},l_{h}\right)}(u|t) & =\frac{S_{ij^{*}}\{u|{ℋ}_{ij^{*}}(u,b_{i}^{\left(l_{m},l_{h}\right)},b_{ij^{*}}^{\left(l_{m},l_{h}\right)},r_{i}^{\left(l_{m},l_{h}\right)},\theta^{\left(l_{m}\right)});\theta^{\left(l_{m}\right)}\}}{S_{ij^{*}}\{t|{ℋ}_{ij^{*}}(t,b_{i}^{\left(l_{m},l_{h}\right)},b_{ij^{*}}^{\left(l_{m},l_{h}\right)},r_{i}^{\left(l_{m},l_{h}\right)},\theta^{\left(l_{m}\right)});\theta^{\left(l_{m}\right)}\}},\\ l_{h} & =1,\ldots ,L_{h}. \end{array}$$
4. Average over $L_{h}$ draws to obtain
$${\bar{\pi }}_{ij^{*}}^{\left(l_{m}\right)}(u|t)=\frac{1}{L_{h}}\overset{L_{h}}{\underset{l_{h}=1}{\sum }}\pi_{ij^{*}}^{\left(l_{m},l_{h}\right)}(u|t)$$
5. The point estimate of $\pi_{ij^{*}}(u|t)$ is obtained by averaging over the Monte Carlo samples:
$${\hat{\pi }}_{ij^{*}}(u|t)=\frac{1}{L_{m}}\overset{L_{m}}{\underset{l_{m}=1}{\sum }}{\bar{\pi }}_{ij^{*}}^{\left(l_{m}\right)}(u|t).$$
  A 95% credible interval is computed using empirical 2.5th and 97.5th percentiles of the Monte Carlo sample ${\bar{\pi }}_{ij^{*}}^{\left(l_{m}\right)}(u|t),\;l_{m}=1,\ldots ,L_{m}$.

Previous work has used a Metropolis Hastings algorithm to sample random effects from the posterior distribution [30, 31] in Step 2. However, because the dimension of $B_{i}$ can be large, this approach can be computationally expensive. We therefore use Stan to sample the random effects. For each posterior draw $\theta^{\left(l_{m}\right)}$, the model parameters are fixed and only the random effects are sampled, which substantially reduces the dimension of sampling and makes the computation feasible in practice.

An additional advantage of making prediction conditional on the full observed history of the cluster is that, in Step 2, we obtain posterior draws for the random effects of all units in the cluster, not only those of the target unit $ij^{*}$. As a result, $\pi_{ij}^{\left(l_{m}\right)}(u|t)$ can be computed simultaneously for all units ij that remain at risk at time t. Thus, by conditioning on information from all units within the cluster, the proposed method is able to make dynamic prediction for all units that survived until time t simultaneously.

### 2.4 Evaluation of Predictive Performance

The predictive performance of the dynamic prediction models is assessed through two measures: discrimination and calibration. Discrimination evaluates how well the model differentiates between units that will experience the event from those that will not [32], whereas calibration measures the agreement between predicted probabilities and observed outcomes [33, 34].

In the context of survival outcome with longitudinal biomarker, discrimination must account for the time‐dynamic nature of the conditional survival probability, $\pi_{ij}(u|t)$. Specifically, we focus on discriminating between units that will experience the event within a time interval (t,u] and those that will not, given the biomarker measurements up to time t. The dynamic AUC is used to quantify the discriminative ability. Dynamic AUC is defined as the probability that, between two randomly selected units, the one with the shorter predicted survival time actually experiences the event earlier [9].

There are two challenges in computing dynamic AUC. First, when we compare the observed survival time of two units, not all unit pairs are comparable (i.e., their observed survival time can be ordered) in the time interval (t,u]. If one or both units are censored in (t,u], their survival time cannot be ordered. To address this, we weight the AUC contributed from non‐comparable pairs by the inverse probability that they would be comparable [35]. Two approaches are considered: a model‐based approach, which uses the fitted joint model for the censoring distribution [9], and an inverse probability of censoring weighting (IPCW) approach, which uses a Kaplan–Meier estimator for the censoring distribution [23, 35]. Second, for clustered data, units within the same cluster are not independent. Thus, AUC calculations need to take group membership into account when comparing pairs.

To address clustering, we distinguish between two types of pairwise comparisons: two units within the same cluster and two units in different clusters. Accordingly, we define three AUC measures: within‐cluster AUC, comparing units within the same cluster; between‐cluster AUC, comparing units from different clusters; and overall AUC, a weighted mean of the within‐cluster AUC and between‐cluster AUC.

For i=1,…,N, $j,j^{\prime }=1,\ldots ,n_{i}$, $j\neq j^{\prime }$ and u>t, the within‐cluster AUC is defined as:

$${\text{AUC}}_{W}(t,u)=\mathrm{Pr}\left[\pi_{ij}(u|t)<\pi_{ij^{\prime }}(u|t)\;|\;\{T_{ij}^{*}\in (t,u]\}\cap \{T_{ij^{\prime }}^{*}>u\}\right].$$

For $i,i^{\prime }=1,\ldots ,N$, $i\neq i^{\prime }$, $j=1,\ldots ,n_{i}$, $j^{\prime }=1,\ldots ,n_{i^{\prime }}$ and u>t, the between‐cluster AUC is defined as:

$${\text{AUC}}_{B}(t,u)=\mathrm{Pr}\left[\pi_{ij}(u|t)<\pi_{i^{\prime }j^{\prime }}(u|t)\;|\;\{T_{ij}^{*}\in (t,u]\}\cap \{T_{i^{\prime }j^{\prime }}^{*}>u\}\right],$$

The overall AUC is then calculated as:

(10) $${\text{AUC}}_{O}(t,u)=\frac{n_{comp,W}}{n_{comp,O}}{\text{AUC}}_{W}(t,u)+\frac{n_{comp,B}}{n_{comp,O}}{\text{AUC}}_{B}(t,u),$$

where $n_{comp,W}$ is the number of within‐cluster comparable pairs, $n_{comp,B}$ is the number of between‐cluster comparable pairs, and $n_{comp,O}$ is the total number of comparable pairs.

To estimate within‐cluster AUC, we compute the average of ${\text{AUC}}_{model,W,i}(t,u)$ across all clusters i=1,…,N. Specifically,

$${\hat{\text{AUC}}}_{W}(t,u)=\frac{1}{N}\overset{N}{\underset{i=1}{\sum }}{\hat{\text{AUC}}}_{W,i}(t,u).$$

We use ${\hat{\text{AUC}}}_{model,W,i}(t,u)$ and ${\hat{\text{AUC}}}_{IPCW,W,i}(t,u)$ to denote the estimator of ${\text{AUC}}_{W,i}(t,u)$ under the model‐based and IPCW approaches, respectively. Under the model‐based approach, each ${\hat{\text{AUC}}}_{model,W,i}(t,u)$ can be decomposed into four components based on comparability:

$$\begin{array}{ll} {\hat{\text{AUC}}}_{model,W,i}(t,u) & ={\hat{\text{AUC}}}_{model,W,i}^{\left(1\right)}(t,u)+{\hat{\text{AUC}}}_{model,W,i}^{\left(2\right)}(t,u)\\ & \;+{\hat{\text{AUC}}}_{model,W,i}^{\left(3\right)}(t,u)+{\hat{\text{AUC}}}_{model,W,i}^{\left(4\right)}(t,u). \end{array}$$

We define the comparable and non‐comparable pairs by

(11) $$\begin{array}{ll} & \Omega_{ij,ij^{\prime }}^{\left(1\right)}(t)=\{T_{ij}\in (t,u],\delta_{ij}=1\}\;\cap \;\{T_{ij^{\prime }}>u\}, \end{array}$$

(12) $$\begin{array}{ll} & \Omega_{ij,ij^{\prime }}^{\left(2\right)}(t)=\{T_{ij}\in (t,u],\delta_{ij}=0\}\;\cap \;\{T_{ij^{\prime }}>u\}, \end{array}$$

(13) $$\begin{array}{ll} & \Omega_{ij,ij^{\prime }}^{\left(3\right)}(t)=\{T_{ij}\in (t,u],\delta_{ij}=1\}\;\cap \;\{T_{ij^{\prime }}\in (T_{ij},u],\delta_{ij^{\prime }}=0\}, \end{array}$$

(14) $$\begin{array}{ll} & \Omega_{ij,ij^{\prime }}^{\left(4\right)}(t)=\{T_{ij}\in (t,u],\delta_{ij}=0\}\;\cap \;\{T_{ij^{\prime }}\in (T_{ij},u],\delta_{ij^{\prime }}=0\}. \end{array}$$

It follows for m=1,2,3,4, ${\hat{\text{AUC}}}_{W,i}^{\left(m\right)}(t,u)$ can be estimated as the proportion of concordant units weighted by the probability of being comparable at time t. Specifically:

$${\hat{\text{AUC}}}_{model,W,i}^{\left(m\right)}(t,u)=\frac{\begin{array}{l} \sum_{j=1}^{n_{i}}\sum_{j^{\prime }=1,j^{\prime }\neq j}^{n_{i}}I\{{\hat{\pi }}_{ij}(u|t)<{\hat{\pi }}_{ij^{\prime }}(u|t)\}\\ \times I\{\Omega_{ij,ij^{\prime }}^{\left(m\right)}(t)\}\times v_{ij,ij^{\prime }}^{\left(m\right)} \end{array}}{\sum_{j=1}^{n_{i}}\sum_{j^{\prime }=1,j^{\prime }\neq j}^{n_{i}}I\{\Omega_{ij,ij^{\prime }}^{\left(m\right)}(t)\}\times v_{ij,ij^{\prime }}^{\left(m\right)}},$$

where I() is an indicator function, and the weights can be estimated using the estimated dynamic survival probabilities:

(15) $$\begin{array}{ll} & {\hat{v}}_{ij,ij^{\prime }}^{\left(1\right)}=1, \end{array}$$

(16) $$\begin{array}{ll} & {\hat{v}}_{ij,ij^{\prime }}^{\left(2\right)}=1-{\hat{\pi }}_{ij}(u|T_{ij}), \end{array}$$

(17) $$\begin{array}{ll} & {\hat{v}}_{ij,ij^{\prime }}^{\left(3\right)}={\hat{\pi }}_{ij^{\prime }}(u|T_{ij^{\prime }}), \end{array}$$

(18) $$\begin{array}{ll} & {\hat{v}}_{ij,ij^{\prime }}^{\left(4\right)}=\{1-{\hat{\pi }}_{ij}(u|T_{ij})\}\times {\hat{\pi }}_{ij^{\prime }}(u|T_{ij;}), \end{array}$$

where $v_{ij,ij^{\prime }}^{\left(2\right)}$ is the probability that unit ij experiences the event in $\left(T_{ij},u\right]$, $v_{ij,ij^{\prime }}^{\left(3\right)}$ is the probability that unit $ij^{\prime }$ survives in $\left(T_{ij^{\prime }},u\right]$, and $v_{ij,ij^{\prime }}^{\left(4\right)}$ is the probability that unit ij experiences the event in $\left(T_{ij},u\right]$ and unit $ij^{\prime }$ survives in $\left(T_{ij^{\prime }},u\right]$.

Under the IPCW approach, the contribution of ${\text{AUC}}_{W,i}(t,u)$ from ith cluster can be estimated by:

$$\begin{array}{ll} & {\hat{\text{AUC}}}_{IPCW,W,i}(t,u)\\ & \;=\frac{\begin{array}{l} \sum_{j=1}^{n_{i}}\sum_{j^{\prime }=1,j^{\prime }\neq j}^{n_{i}}I\{{\hat{\pi }}_{ij}(u|t)<{\hat{\pi }}_{ij^{\prime }}(u|t)\}\\ {\tilde{D}}_{ij}(t,u)(1-{\tilde{D}}_{ij^{\prime }}(t,u)){Ŵ}_{ij}(t,u){Ŵ}_{ij^{\prime }}(t,u) \end{array}}{\sum_{j=1}^{n_{i}}\sum_{j^{\prime }=1,j^{\prime }\neq j}^{n_{i}}{\tilde{D}}_{ij}(t,u)(1-{\tilde{D}}_{ij^{\prime }}(t,u)){Ŵ}_{ij}(t,u){Ŵ}_{ij^{\prime }}(t,u)}, \end{array}$$

where ${\tilde{D}}_{ij}(t,u)=I(t<T_{ij}\leq u,\delta_{ij}=1)$, and the weights are constructed using the Kaplan–Meier estimate of the censoring distribution:

(19) $${Ŵ}_{ij}(t,u)=\frac{I(T_{ij}>u)}{Ĝ(u|t)}+\frac{I(t<T_{ij}\leq u)\delta_{ij}}{Ĝ(T_{ij}|t)},$$

with Ĝ() denoting the Kaplan‐Meier estimate of the censoring distribution.

The model‐based estimator of between‐cluster AUC can be decomposed analogously into four components based on comparability:

$$\begin{array}{ll} {\hat{\text{AUC}}}_{model,B}(t,u) & ={\hat{\text{AUC}}}_{model,B}^{\left(1\right)}(t,u)+{\hat{\text{AUC}}}_{model,B}^{\left(2\right)}(t,u)\\ & \;+{\hat{\text{AUC}}}_{model,B}^{\left(3\right)}(t,u)+{\hat{\text{AUC}}}_{model,B}^{\left(4\right)}(t,u). \end{array}$$

For m=1,2,3,4, we have

$$\begin{array}{ll} & {\hat{\text{AUC}}}_{model,B}^{\left(m\right)}(t,u)\\ & \;=\frac{\begin{array}{l} \sum_{i=1}^{N}\sum_{j=1}^{n_{i}}\left[\sum_{i^{\prime }=1,i^{\prime }\neq i}^{N}\sum_{j^{\prime }=1}^{n_{i^{\prime }}}I\{{\hat{\pi }}_{ij}(u|t)<{\hat{\pi }}_{i^{\prime }j^{\prime }}(u|t)\}\right.\\ \left.\times I\{\Omega_{ij,i^{\prime }j^{\prime }}^{\left(m\right)}(t)\}\times {\hat{v}}_{ij,i^{\prime }j^{\prime }}^{\left(m\right)}\right] \end{array}}{\sum_{i=1}^{N}\sum_{j=1}^{n_{i}}\left[\sum_{i^{\prime }=1,i^{\prime }\neq i}^{N}\sum_{j^{\prime }=1}^{n_{i^{\prime }}}I\{\Omega_{ij,i^{\prime }j^{\prime }}^{\left(m\right)}(t)\}\times {\hat{v}}_{ij,i^{\prime }j^{\prime }}^{\left(m\right)}\right]}, \end{array}$$

where the pairs $\Omega_{ij,i^{\prime }j^{\prime }}^{\left(m\right)}(t)$ are defined similarly as in Equations (11) to (14), and $v_{ij,i^{\prime }j^{\prime }}^{\left(m\right)}$ are estimated based on Equations (15) to (18).

Similarly, the IPCW between‐cluster AUC can be estimated by

$$\begin{array}{ll} & {\hat{\text{AUC}}}_{IPCW,B}(t,u)\\ & \;=\frac{\begin{array}{l} \sum_{i=1}^{N}\sum_{j=1}^{n_{i}}\left[\sum_{i^{\prime }=1,i^{\prime }\neq i}^{N}\sum_{j^{\prime }=1}^{n_{i^{\prime }}}I\{{\hat{\pi }}_{ij}(u|t)<{\hat{\pi }}_{i^{\prime }j^{\prime }}(u|t)\}\right.\\ \left.{\tilde{D}}_{ij}(t,u)(1-{\tilde{D}}_{i^{\prime }j^{\prime }}(t,u)){Ŵ}_{ij}(t,u){Ŵ}_{i^{\prime }j^{\prime }}(t,u)\right] \end{array}}{\begin{array}{l} \sum_{i=1}^{N}\sum_{j=1}^{n_{i}}\left[\sum_{i^{\prime }=1,i^{\prime }\neq i}^{N}\sum_{j^{\prime }=1}^{n_{i^{\prime }}}{\tilde{D}}_{ij}(t,u)(1-{\tilde{D}}_{i^{\prime }j^{\prime }}(t,u))\right.\\ \left.{Ŵ}_{ij}(t,u){Ŵ}_{i^{\prime }j^{\prime }}(t,u)\right]. \end{array}} \end{array}$$

Finally, the overall AUC, ${\text{AUC}}_{O}(t,u)$, is estimated by substituting the estimated model‐based or IPCW ${\hat{\text{AUC}}}_{W}(t,u)$ and ${\hat{\text{AUC}}}_{B}(t,u)$ into Equation (10).

Calibration measures how well the predicted probabilities of the model align with the observed outcomes [33, 34]. It can be evaluated using the expected quadratic prediction error (PE). In our setting, we incorporate the longitudinal biomarker and focus on predicting the occurrence of event for a unit at time u given measurements up to time t [36]. The PE is defined as:

$$\text{PE}(u|t)=E[{\left\{I(T_{ij}^{*}>u)-\pi_{ij}(u|t)\right\}}^{2}].$$

To account for censoring, again, both model‐based and IPCW approaches are considered. Following Henderson [37], a model‐based estimate of PE(u|t) is given by

$$\begin{array}{ll} {\hat{\text{PE}}}_{model}(u|t) & =\frac{1}{R(t)}\underset{ij:T_{ij}>t}{\sum }I(T_{ij}>u){\left\{1-{\hat{\pi }}_{ij}(u|t)\right\}}^{2}\\ & \;+\delta_{ij}I(T_{ij}\leq u){\left\{0-{\hat{\pi }}_{ij}(u|t)\right\}}^{2}\\ & \;+(1-\delta_{ij})I(T_{ij}\leq u)\left[{\hat{\pi }}_{ij}(u|T_{ij}){\left\{1-{\hat{\pi }}_{ij}(u|t)\right\}}^{2}\right.\\ & \;\left.+\{1-{\hat{\pi }}_{ij}(u|T_{ij})\}{\left\{0-{\hat{\pi }}_{ij}(u|t)\right\}}^{2}\right], \end{array}$$

where R(t) is the number of units at risk at time t. In the summation, the first term corresponds to units that remain event‐free in (t,u]; the second term corresponds to units that experience the event in (t,u]; and the third term corresponds to units that are interval‐censored in (t,u], for whom we introduce the model‐based probability weight of event‐free or experiencing the event in $\left(T_{ij},u\right]$.

The IPCW PE estimator is given by

$${\hat{\text{PE}}}_{IPCW}(u|t)=\frac{1}{R(t)}\underset{ij:T_{ij}>t}{\sum }{Ŵ}_{ij}(t,u){\left\{I(T_{ij}\leq u)-{\hat{\pi }}_{ij}(u|t)\right\}}^{2},$$

where the observations are weighted by the inverse probability of remaining uncensored, estimated via the Kaplan Meier estimator given by Equation (19).

A detailed comparison of model‐based and IPCW approaches under the joint model framework is provided by Rizopoulos and Taylor [24]. In general, the model‐based approach yields valid estimates when censoring depends on longitudinal history or baseline covariates, but it relies on correct specification of the joint model. The IPCW approach is more robust to model misspecification but assumes that censoring is independent of the longitudinal process and covariates. Therefore, the choice between these approaches should be guided by the underlying data‐generating mechanism and the plausibility of their respective assumptions.

## 3 Simulation Study

### 3.1 Simulation Design

We conducted simulation studies to assess the finite sample performance of our proposed method. The simulated data were generated from a joint model with two longitudinal submodels, one for the continuous outcome and one for the binary outcome, and a survival submodel. Each longitudinal submodel included biomarker‐specific random effects at cluster‐level and unit‐level. The baseline hazard of the survival submodel was generated from a Weibull distribution.

We simulated two time‐independent covariates. The cluster‐level continuous variable $X_{i}$ was generated from $X_{i}∼N(0,1)$, and the unit‐level binary variable $Z_{ij}$ was generated from $Z_{ij}∼\text{Bernoulli}(0.3)$. From these, the longitudinal outcomes for the continuous and binary biomarkers were generated from

$$\begin{array}{ll} Y_{Aij}(t) & =\eta_{Aij}(t)+\epsilon_{ij}(t)=1.3+0.1X_{i}-0.1t\\ & \;+0.3Z_{ij}+b_{Ai}+b_{Aij,0}+b_{Aij,1}t+\epsilon_{ij}(t), \end{array}$$

$$\begin{array}{ll} \text{logit}(E(Y_{Bij}(t))) & =\eta_{Bij}(t)=-0.8-X_{i}+0.2t-1.4Z_{ij}\\ & \;+b_{Bi}+b_{Bij,0}+b_{Bij,1}t, \end{array}$$

and the hazard for the terminal event was generated from

$$\begin{array}{ll} h_{ij}(t) & =t^{\xi -1}\times \exp(-6+r_{i}+0.1X_{i}+1.7Z_{ij}\\ & \;+4\eta_{Aij}(t)+0.13\eta_{Bij}(t)), \end{array}$$

where $\epsilon_{ij}(t)∼N(0,\sigma_{e}^{2})$, $r_{i}∼N(0,\sigma_{r}^{2})$, $b_{i}={\left(b_{Ai},b_{Bi}\right)}^{T}∼MVN(0,\sum_{C})$ and $b_{\mathrm{ij}}={\left(b_{Aij,0},b_{Aij,1},b_{Bij,0},b_{Bij,1}\right)}^{T}∼MVN(0,\sum_{U})$. The shape parameter of the baseline hazard was ξ=0.8; the standard deviations of the residual error and frailty term were $\left(\sigma_{e},\sigma_{r})=(0.2,0.6\right)$; the standard deviations in the covariance matrix of the cluster‐level random effects were $\left(\sigma_{CA},\sigma_{CB})=(0.15,4\right)$ with the correlation parameter $\rho_{C}=0.5$; and the standard deviations in the unit‐level random effects were $\left(\sigma_{UA0},\sigma_{UA1},\sigma_{UB0},\sigma_{UB1})=(0.2,0.03,6,0.6\right)$ with the correlation parameters $\left(\rho_{1},\rho_{2},\rho_{3},\rho_{4},\rho_{5},\rho_{6})=(-0.5,0.2,0.1,0.1,0.3,-0.4\right)$. The true values of the parameters were determined based on the result of real data analysis presented in Section 4.

We set the number of cluster as N=100. Within each cluster, the number of units was generated from $n_{i}∼\text{uniform}(22,28)$. We assumed that each cluster was observed every 6 months with a total of 16 follow‐up visits. The censoring time for the terminal event was 8 years, which ensured a censoring rate of approximately 80%. To generate the data, we first simulated the cluster sizes, covariates, and random effects. Conditional on these, we generated the terminal event time and the biomarker values at each follow‐up time.

For each simulated data set, we fitted the proposed multilevel joint model and compared it with two existing approaches: a joint model implemented in the JMbayes2 package [31] and a Stan joint model (Stan JM) [17] implemented in the rstanarm package [38]. Although both JMbayes2 and Stan JM allow multiple longitudinal biomarkers, they do not support multilevel data structure. Therefore, their longitudinal submodels included only unit‐level random intercepts and slopes, assuming independence between units within clusters. The survival submodel in JMbayes2 was specified using a Cox proportional hazard model with a B‐spline baseline hazard, while Stan JM assumed a Weibull baseline hazard. Since JMbayes2 and Stan JM cannot incorporate multilevel random effects, we focused on comparing the fixed‐effect parameter estimates.

We repeated the simulation 1000 times. We calculated the mean estimates (M‐Est), relative bias (Rel‐Bias) as the ratio of average bias to the true value, the mean of the posterior standard deviation (M‐SD), the 95% credible intervals (95% CI) determined by the 0.025th and 0.975th quantiles of the posterior distribution of the parameters, and the coverage probability (Coverage) as the proportion of times the 95% CI contained the true value.

To evaluate dynamic prediction performances, we considered three scenarios when correlations of survival outcomes within the same cluster are small, moderate, and large, by varying the standard deviations of frailty term with $\sigma_{r}\in \{0.2,0.6,1.2\}$. For each scenario, we performed an external validation procedure: from each simulated data set, we randomly selected 50 clusters as the training set and used the remaining 50 clusters as the testing set. Since dynamic risk prediction is not implemented in rstanarm, we focused on comparing the proposed multilevel joint model and the joint model from JMbayes2. Models were fitted to the training set, and risk predictions were evaluated on the testing set. We compared three prediction approaches: (i) cluster‐informed prediction from the proposed multilevel joint model, (ii) unit‐informed prediction using the specific unit's longitudinal history from the proposed multilevel joint model, and (iii) unit‐informed prediction from JMbayes2. Predictive performance was assessed using time‐varying overall AUC, ${\text{AUC}}_{O}(t,u)$, and prediction error, PE(u|t), based on model‐based and IPCW estimators. The landmark time was fixed at t=4, with prediction horizons u∈{4.5,5,5.5,6}.

### 3.2 Simulation Results

The simulation results of model fitting of the proposed multilevel joint model and the JMbayes2 model are summarized in Table 1. A more detailed comparison with Stan JM is provided in Table S1 in the Supporting Information. We found that our proposed method performed well, with mean estimates close to the true values and negligible relative bias across all parameters. The corresponding coverage probabilities were close to 95%, indicating that our proposed method estimated appropriate standard errors and credible intervals.

**TABLE 1:** Results from the simulation study of model fitting.

		Multilevel JM				JMbayes2			
Parameter	True	M‐Est	Rel‐Bias	M‐SD	Coverage	M‐Est	Rel‐Bias	M‐SD	Coverage
*Survival submodel*									
$\gamma_{0}$	−6.00	−6.084	0.014	0.203	0.912				
$\gamma_{1}$	0.10	0.087	−0.129	0.075	0.954	0.085	−0.150	0.040	0.750
$\gamma_{2}$	1.70	1.675	−0.015	0.092	0.951	1.527	−0.102	0.083	0.460
$\alpha_{1}$	4.00	4.068	0.017	0.154	0.922	3.656	−0.086	0.182	0.508
$\alpha_{2}$	0.13	0.130	−0.002	0.012	0.944	0.121	−0.068	0.008	0.751
*Longitudinal submodel (continuous biomarker)*									
$\beta_{AX0}$	1.30	1.295	−0.004	0.024	0.952	1.300	0.000	0.007	0.600
$\beta_{AX1}$	0.10	0.102	0.016	0.018	0.945	0.100	0.002	0.005	0.480
$\beta_{AX2}$	−0.10	−0.100	0.003	0.002	0.959	−0.100	0.000	0.002	0.938
$\beta_{AZ1}$	0.30	0.300	−0.001	0.016	0.945	0.302	0.007	0.013	0.963
$\sigma_{e}$	0.20	0.204	0.020	0.008	0.959	0.200	0.000	0.001	0.971
*Longitudinal submodel (binary biomarker)*									
$\beta_{BX0}$	−0.80	−0.757	−0.054	0.438	0.956	−0.773	−0.034	0.203	0.621
$\beta_{BX1}$	−1.00	−0.966	−0.034	0.433	0.956	−0.999	−0.001	0.172	0.561
$\beta_{BX2}$	0.20	0.198	−0.011	0.039	0.952	0.203	0.015	0.039	0.947
$\beta_{BZ1}$	−1.40	−1.381	−0.014	0.354	0.961	−1.340	−0.043	0.397	0.974
*Random effects*									
$\sigma_{r}$	0.60	0.611	0.018	0.059	0.954				
$\sigma_{CA}$	0.15	0.153	0.023	0.018	0.944				
$\sigma_{CB}$	4.00	4.037	0.009	0.371	0.936				
$\rho_{C}$	0.50	0.472	−0.055	0.089	0.952				
$\sigma_{UA0}$	0.20	0.203	0.013	0.015	0.945	0.249	0.245	0.006	0.004
$\sigma_{UA1}$	0.03	0.036	0.211	0.012	0.956	0.030	0.004	0.002	0.987
$\sigma_{UB0}$	6.00	6.029	0.005	0.289	0.940	7.169	0.195	0.353	0.082
$\sigma_{UB1}$	0.60	0.609	0.015	0.040	0.945	0.603	0.005	0.036	0.913
$\rho_{1}$	−0.50	−0.490	−0.020	0.042	0.938	−0.397	−0.206	0.043	0.384
$\rho_{2}$	0.20	0.192	−0.039	0.043	0.947	0.298	0.488	0.026	0.133
$\rho_{3}$	0.10	0.104	0.045	0.069	0.933	0.102	0.017	0.073	0.943
$\rho_{4}$	0.10	0.106	0.061	0.060	0.944	0.081	−0.190	0.053	0.892
$\rho_{5}$	0.30	0.300	−0.001	0.072	0.956	0.307	0.025	0.066	0.981
$\rho_{6}$	−0.40	−0.379	−0.053	0.070	0.949	−0.275	−0.313	0.105	0.822

*Note:* Multilevel JM denotes the proposed joint model for multilevel data. JMbayes2 refers to a joint model from JMbayes2 package, assuming independence between units within each cluster M‐Est is the mean of parameter estimates; Rel‐Bias is the ratio of average bias to the true value; M‐SD is the mean of the posterior standard deviation; and Coverage is the empirical coverage probability as the proportion of times the 95% credible interval contains the true value.

For the JMbayes2 model, we observed undercoverage in the survival submodel parameters, primarily driven by bias. The relative biases for the survival parameters from the Stan JM were generally larger. For both JMbayes2 and Stan JM, the fixed‐effect estimates in the longitudinal submodels exhibited small relative bias; however, the observed undercoverage is caused by underestimated standard errors.

For each simulated data set, we ran four parallel chains with 2000 iterations per chain, discarding the first 1000 iterations as burn‐in. In terms of computation time, using a MacBook Pro with an Apple M2 Pro chip and 32 GB of RAM, fitting the proposed multilevel joint model to a dataset with 100 clusters and approximately 2500 units required about 1 h. In comparison, the JMbayes2 model took approximately 2 min, while the Stan JM took about 3 h and 30 min.

**FIGURE 2:**
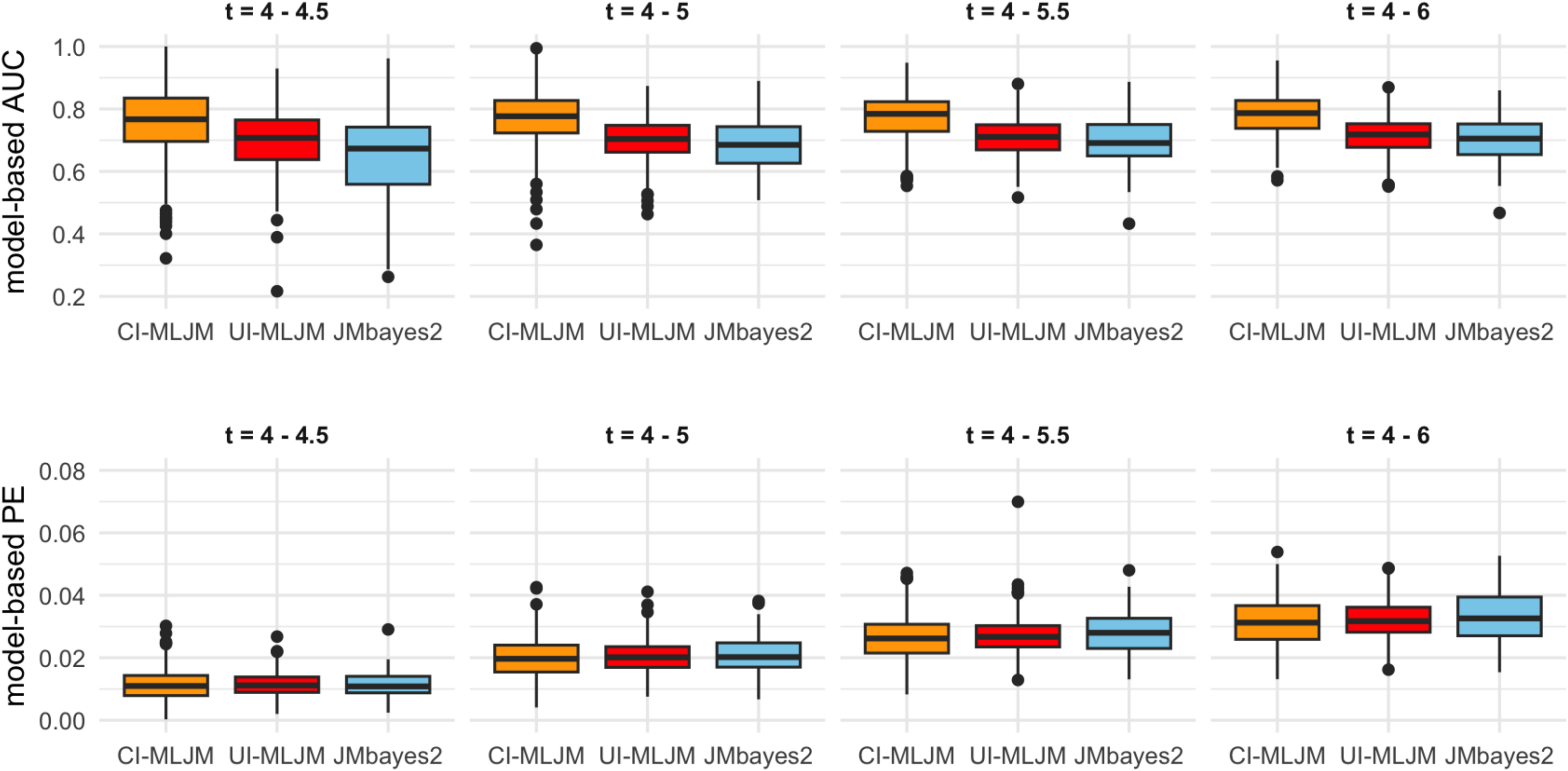
Boxplots of model‐based time‐varying overall AUC and PE across different prediction windows, with the true standard deviation of frailty term assumed to be 1.2. The bold middle line represents the median. The lower and upper hinges correspond to the 25th and 75th percentiles. CI‐MLJM denotes the cluster‐informed prediction from the proposed multilevel joint model. UI‐MLJM denotes the unit‐informed prediction from the proposed multilevel joint model. JMbayes2 refers to unit‐informed prediction from JMbayes2 package, which assumes independence between units within each cluster.

**FIGURE 3:**
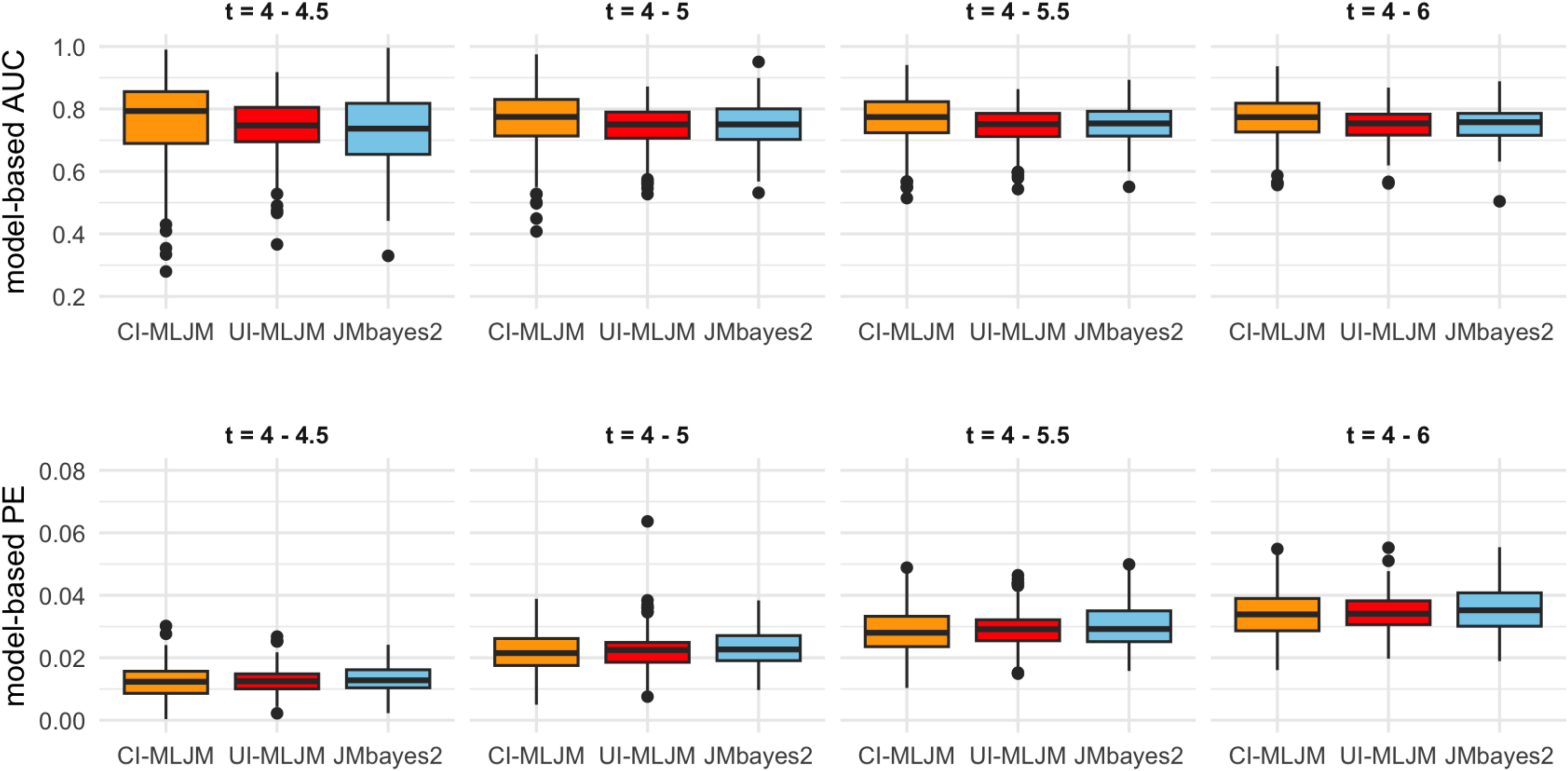
Boxplots of model‐based time‐varying overall AUC and PE across different prediction windows, with the true standard deviation of frailty term assumed to be 0.6. The bold middle line represents the median. The lower and upper hinges correspond to the 25th and 75th percentiles. CI‐MLJM denotes the cluster‐informed prediction from the proposed multilevel joint model. UI‐MLJM denotes the unit‐informed prediction from the proposed multilevel joint model. JMbayes2 refers to unit‐informed prediction from JMbayes2 package, which assumes independence between units within each cluster.

**FIGURE 4:**
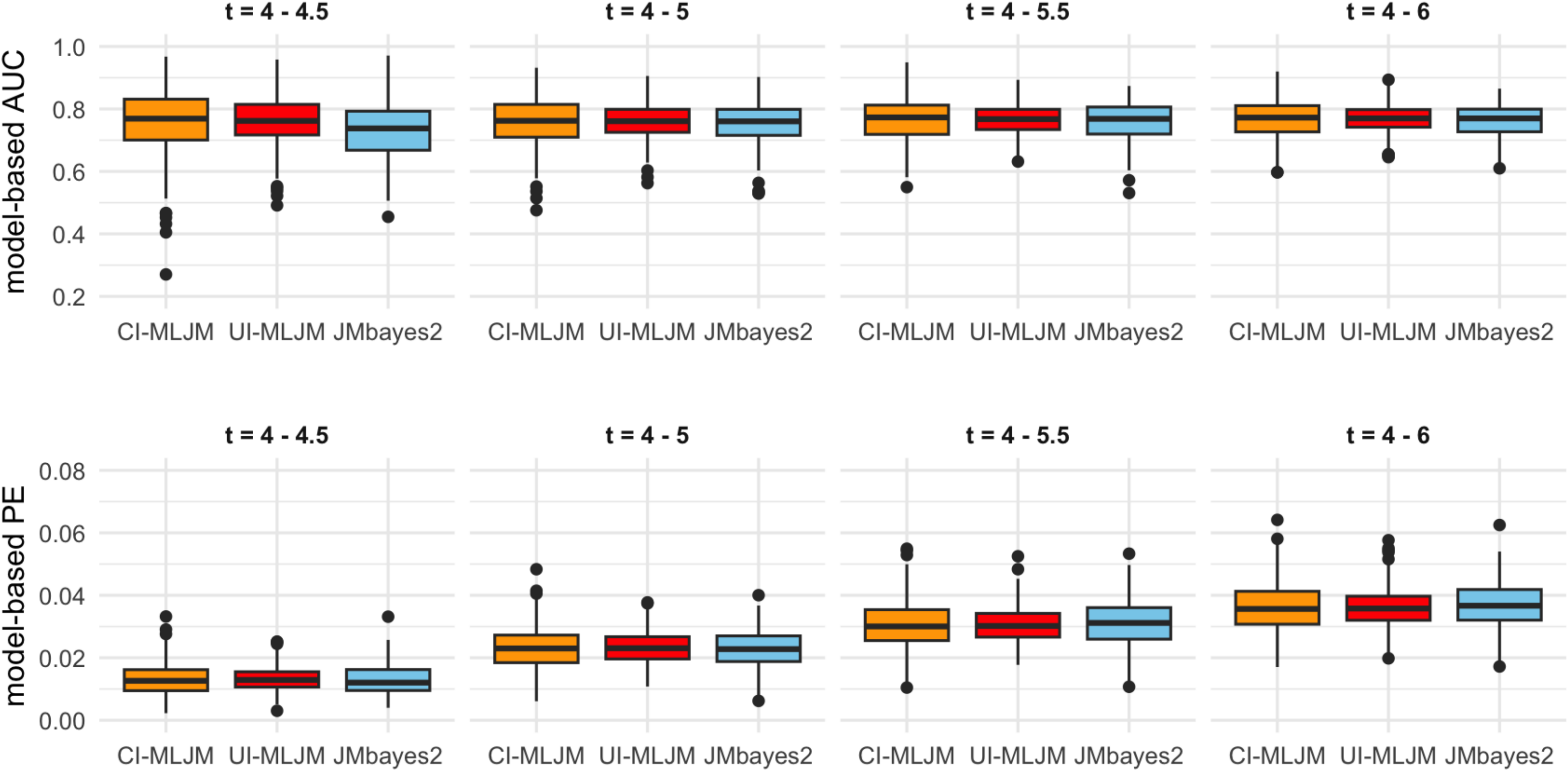
Boxplots of model‐based time‐varying overall AUC and PE across different prediction windows, with the true standard deviation of frailty term assumed to be 0.2. The bold middle line represents the median. The lower and upper hinges correspond to the 25th and 75th percentiles. CI‐MLJM denotes the cluster‐informed prediction from the proposed multilevel joint model. UI‐MLJM denotes the unit‐informed prediction from the proposed multilevel joint model. JMbayes2 refers to unit‐informed prediction from JMbayes2 package, which assumes independence between units within each cluster.

Figures 2, 3, 4 show boxplots of model‐based estimates of AUC and PE, comparing three approaches: (i) cluster‐informed prediction from the proposed multilevel joint model, (ii) unit‐informed prediction from the proposed model, and (iii) unit‐informed prediction from JMbayes2, under the scenarios where the true frailty standard deviations are $\sigma_{r}=1.2,0.6,0.2$, respectively. In the Supporting Information, Figures S1–S3 provide additional results for IPCW estimates of AUC and PE when $\sigma_{r}=1.2,0.6,0.2$. Across all prediction windows, the cluster‐informed prediction approach consistently achieved higher AUCs and lower PEs under both model‐based and IPCW methods, comparing to the unit‐informed prediction from proposed model and JMbayes2. Moreover, when $\sigma_{r}$ is small, the differences in AUCs across all models are negligible. In contrast, as $\sigma_{r}$ increases, the difference of AUCs across all models become more pronounced.

## 4 Data Application

We applied our proposed joint model to electronic periodontal data obtained from the Canadian Armed Forces (CAF). Members of the CAF received comprehensive preventive dental care, and those who required a periodontal exam were referred to a periodontologist. There were 8382 actively serving members of the CAF that received at least one comprehensive periodontal exam between 2007 January 1st and 2021 December 31st. Among these, 2048 subjects had exams where both PPD and mobility were available. We further restricted our analysis to 936 subjects who had at least 5 follow‐up visits. The number of teeth for each subject ranged from 15 to 28 at baseline. The maximum total number of teeth in an adult was 28 since we excluded the third molars (wisdom teeth) from the study sample. In total, there were 24 888 teeth at baseline, and 731 of them were lost during follow‐up. The number of follow‐up visits for each subject ranged from 5 to 22.

Demographic information for each individual was recorded at baseline, including age, sex, and smoking status. The mean baseline age was 41.3 years, with a standard deviation of 7.1 years. Out of the 936 subjects, 812 (86.8%) were male, and 124 (13.2%) were female; 264 (28.2%) were smokers, and 672 (71.8%) were nonsmokers. Out of 24 888 teeth at the baseline, 6848 (27.5%) were molar teeth (the large teeth in the back of the mouth), and 18,040 (72.5%) were non‐molars; 12,519 (50.3%) were mandibular teeth (lower teeth), and 12,369 (49.7%) were maxillary teeth (upper teeth).

In our proposed multilevel joint model, we considered the following cluster‐level covariates: age at baseline (subject's age standardized by the mean and standard deviation), sex (1 for male and 0 for female), smoking status (1 for smoker and 0 for non‐smoker), time from baseline; and the following unit‐level covariates: molar (1 for molar and 0 for non‐molar), and mandibular (1 for mandibular and 0 for maxillary). We assumed a Weibull distribution for the baseline hazard rate. The linear predictors for the log‐transformed PPD ($\eta_{Aij}(t)$) and the logit‐transformed mobility ($\eta_{Bij}(t)$), and the hazard model for the terminal event were modeled as:

$$\begin{array}{ll} \eta_{Aij}(t) & =\beta_{AX0}+\beta_{AX1}{\text{age}}_{i}+\beta_{AX2}{\text{sex}}_{i}+\beta_{AX3}{\text{smoke}}_{i}+\beta_{AX4}t\\ & \;+\beta_{AZ1}{\text{molar}}_{ij}+\beta_{AZ2}{\text{mandibular}}_{ij}+b_{Ai}\\ & \;+b_{Aij,0}+b_{Aij,1}t,\\ \eta_{Bij}(t) & =\beta_{BX0}+\beta_{BX1}{\text{age}}_{i}+\beta_{BX2}{\text{sex}}_{i}+\beta_{BX3}{\text{smoke}}_{i}+\beta_{BX4}t\\ & \;+\beta_{BZ1}{\text{molar}}_{ij}+\beta_{BZ2}{\text{mandibular}}_{ij}+b_{Bi}\\ & \;+b_{Bij,0}+b_{Bij,1}t,\\ h_{ij}(t) & =t^{\xi -1}\times \exp(r_{i}+\gamma_{0}+\gamma_{X1}{\text{age}}_{i}+\gamma_{X2}{\text{sex}}_{i}+\gamma_{X3}{\text{smoke}}_{i}\\ & \;+\gamma_{AZ1}{\text{molar}}_{ij}+\gamma_{AZ2}{\text{mandibular}}_{ij}\\ & \;+\alpha_{1}\eta_{Aij}(t)+\alpha_{2}\eta_{Bij}(t)). \end{array}$$

We compared the proposed multilevel joint model with a joint model from the JMbayes2 package, which specifies the baseline hazard using a B‐spline function. The rstanarm package was not included in the comparison because it is computationally demanding, has a similar model framework with JMbayes2, and does not support dynamic prediction. Table 2 shows the posterior means, posterior standard deviations (SDs), and 95% credible intervals (CIs) for all model parameters based on the CAF data.

**TABLE 2:** Results from the analysis of CAF data.

	Multilevel JM			JMbayes2		
Parameter	Est	SD	95% CI	Est	SD	95% CI
*Survival submodel*						
sex (male = 1)	−0.273	0.178	(−0.624, 0.075)	−**0.341**	0.137	(−0.598, −0.072)
age (standardized)	**0.287**	0.062	(0.169, 0.417)	**0.293**	0.043	(0.210, 0.378)
smoke (yes = 1)	−0.050	0.139	(−0.318, 0.224)	0.041	0.078	(−0.117, 0.185)
molar (yes = 1)	**0.273**	0.106	(0.078, 0.486)	**0.226**	0.094	(0.045, 0.416)
mandibular (yes = 1)	−0.001	0.077	(−0.147, 0.143)	−0.016	0.075	(−0.166, 0.132)
$\alpha_{1}$ (PPD)	**4.062**	0.236	(3.607, 4.534)	**3.925**	0.182	(3.558, 4.275)
$\alpha_{2}$ (Mobility)	**0.057**	0.005	(0.046, 0.067)	**0.057**	0.005	(0.047, 0.066)
*Longitudinal submodel (PPD)*						
intercept	**1.180**	0.012	(1.156, 1.203)	**1.177**	0.004	(1.169, 1.186)
sex (male = 1)	**0.061**	0.013	(0.037, 0.087)	**0.062**	0.004	(0.054, 0.069)
age (standardized)	−**0.009**	0.004	(−0.017, −0.001)	−**0.010**	0.001	(−0.012, −0.007)
smoke (yes = 1)	**0.081**	0.010	(0.062, 0.100)	**0.080**	0.003	(0.074, 0.086)
time (years)	−**0.005**	0.000	(−0.006, −0.005)	−**0.005**	0.000	(−0.006, −0.005)
molar (yes = 1)	**0.309**	0.002	(0.304, 0.314)	**0.310**	0.003	(0.304, 0.316)
mandibular (yes = 1)	−**0.064**	0.002	(−0.068, −0.060)	−**0.062**	0.003	(−0.068, −0.057)
$\sigma_{e}$	**0.192**	0.000	(0.191, 0.193)	**0.192**	0.000	(0.191, 0.193)
*Longitudinal submodel (Mobility)*						
intercept	−**8.306**	0.622	(−9.502, −7.048)	−**6.455**	0.310	(−7.045, −5.844)
sex (male = 1)	−**1.993**	0.629	(−3.237, −0.781)	−**1.834**	0.241	(−2.305, −1.367)
age (standardized)	**1.024**	0.201	(0.661, 1.425)	**0.890**	0.079	(0.736, 1.045)
smoke (yes = 1)	**1.623**	0.486	(0.653, 2.566)	**1.498**	0.177	(1.154, 1.844)
time (years)	**0.325**	0.019	(0.287, 0.364)	**0.074**	0.019	(0.033, 0.112)
molar (yes = 1)	−**3.062**	0.199	(−3.450, −2.680)	−**2.761**	0.203	(−3.161, −2.370)
mandibular (yes = 1)	−**1.846**	0.164	(−2.176, −1.538)	−**1.692**	0.172	(−2.035, −1.360)
*Random effects*						
$\sigma_{r}$	**1.185**	0.077	(1.041, 1.337)			
$\sigma_{CA}$	**0.130**	0.003	(0.124, 0.137)			
$\sigma_{CB}$	**5.897**	0.191	(5.541, 6.266)			
$\rho_{C}$	**0.382**	0.033	(0.318, 0.444)			
$\sigma_{UA0}$	**0.180**	0.001	(0.177, 0.183)	**0.218**	0.002	(0.215, 0.222)
$\sigma_{UA1}$	**0.0220**	0.000	(0.021, 0.022)	**0.022**	0.000	(0.021, 0.022)
$\sigma_{UB0}$	**10.809**	0.221	(10.393, 11.237)	**9.880**	0.302	(9.215, 10.312)
$\sigma_{UB1}$	**0.8159**	0.017	(0.783, 0.848)	**0.723**	0.020	(0.678, 0.752)
$\rho_{1}$	−**0.514**	0.010	(−0.533, −0.495)	−**0.383**	0.010	(−0.401, −0.361)
$\rho_{2}$	**0.180**	0.011	(0.160, 0.203)	**0.240**	0.009	(−0.401, −0.361)
$\rho_{3}$	−**0.070**	0.017	(−0.101, −0.036)	**0.073**	0.016	(0.223, 0.258)
$\rho_{4}$	−**0.119**	0.015	(−0.149, −0.091)	−**0.130**	0.013	(−0.154, −0.106)
$\rho_{5}$	**0.2269**	0.020	(0.190, 0.266)	**0.209**	0.020	(0.171, 0.244)
$\rho_{6}$	−**0.510**	0.022	(−0.551, −0.467)	−**0.120**	0.027	(−0.161, −0.056)

*Note:* Multilevel JM denotes the proposed joint model for multilevel data. JMbayes2 refers to a joint model from JMbayes2 package, assuming independence between units within each cluster. Est is the mean of the posterior distribution, SD is the standard deviation of the posterior distribution, and 95% CI is the 95% credible interval.

In the survival submodel of the proposed multilevel joint model, both age and molar status were significantly associated with the hazard of tooth loss. Specifically, the hazard ratio for age was exp(0.287)=1.332 with 95% CI [1.184, 1.517], and the hazard ratio for molar was exp(0.273)=1.314 with 95% CI [1.081, 1.626]. In addition, both log PPD and log‐odds of mobility were positively associated with the hazard of tooth loss. The JMbayes2 model produced similar estimates for the fixed effects. However, most of the estimated standard deviations from the JMbayes2 model were smaller than those from the proposed joint model, consistent with the findings from the simulation study.

In the longitudinal submodels, the estimated covariate effects from the proposed multilevel joint model were generally similar to those from JMbayes2. Smoking was associated with higher PPD values and a greater probability of mobility. Molar teeth tended to have higher PPD but a lower probability of mobility compared with non‐molar teeth, while mandibular teeth exhibited lower PPD and a lower probability of mobility compared with maxillary teeth. As in the survival submodel, JMbayes2 generally produced smaller estimates of variability, which is again consistent with the simulation results.

Table 3 presents the model‐based and IPCW estimates of AUC and PE for both the cluster‐informed prediction from the proposed multilevel joint model and the unit‐informed prediction from JMbayes2 model. The proposed multilevel joint model achieved higher time‐varying AUCs than the JMbayes2 model, indicating improved discriminative ability in distinguishing teeth at higher versus lower risk of loss, while the corresponding differences in PEs between the two approaches were negligible. In the Supporting Information, Figure S4 provides additional results for the time‐varying Receiver Operating Characteristic (ROC) curves across different prediction windows. The higher time‐varying ROC curves of the multilevel joint model indicated improved discriminative performance across a range of risk thresholds within each prediction window.

**TABLE 3:** Comparison of predictive performance between the cluster‐informed prediction from the proposed multilevel joint model (CI‐Multilevel JM) and unit‐informed prediction from JMbayes2 across different prediction windows for the CAF data.

	Model‐based AUC		Model‐based PE	
Prediction windows	CI‐Multilevel JM	JMbayes2	CI‐Multilevel JM	JMbayes2
(4,4.5]	0.769	0.540	0.002	0.002
(4,5]	0.803	0.619	0.004	0.004
(4,5.5]	0.832	0.700	0.005	0.005
(4,6]	0.837	0.766	0.007	0.007
	**IPCW AUC**		**IPCW PE**	
**Prediction window**	**CI‐Multilevel JM**	**JMbayes2**	**CI‐Multilevel JM**	**JMbayes2**
(4,4.5]	0.734	0.542	0.002	0.002
(4,5]	0.819	0.621	0.004	0.004
(4,5.5]	0.830	0.703	0.005	0.005
(4,6]	0.835	0.773	0.007	0.007

*Note:* Results are summarized using model‐based and IPCW estimators of the time‐varying overall AUC and PE.

**FIGURE 5:**
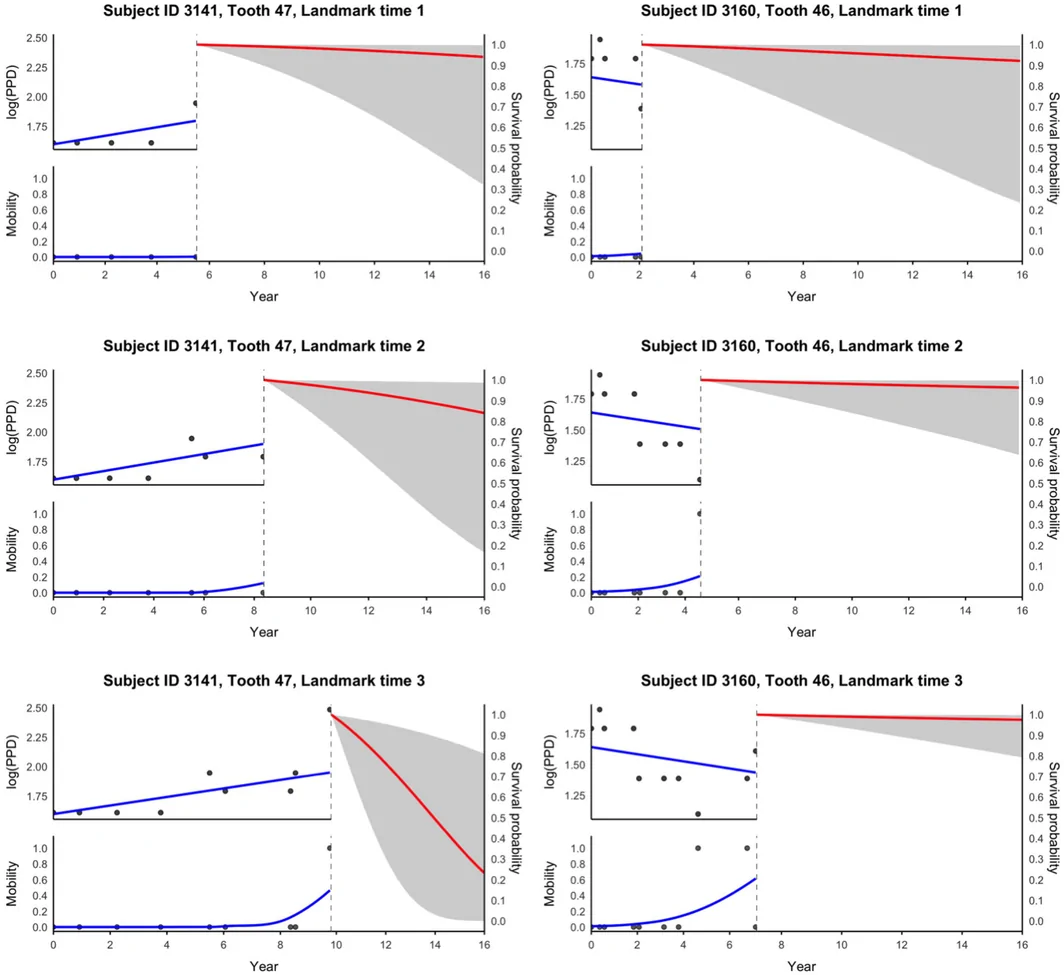
Cluster‐informed dynamic survival predictions of tooth survival for two subjects. The vertical dotted lines indicate landmark times for prediction. On the left, the fitted longitudinal trajectories of log(PPD) and mobility are presented. On the right side, the solid line represents the predicted survival probability, with the shaded region representing the 95% credible interval.

We performed cluster‐informed dynamic predictions of tooth survival for two randomly selected subjects. Figure 5 presents the predicted survival probabilities for two teeth of two subjects at three landmark times, after the most recent clinical visit. For tooth 47 of Subject 3141, the predicted survival probability remained high at landmark times 1 and 2, staying above 0.8 by year 16. However, at landmark time 3, the survival probability decreased more rapidly, falling below 0.3 by year 16. This can be explained by the biomarker history: the tooth exhibited sharp increases in PPD and became mobile at the last visit, indicating a worsening prognosis. For tooth 46 of Subject 3160, the PPD measurements decreased over time and the mobility trajectory remained relatively stable. Consequently, the predicted survival probability remained close to 1 across all prediction windows. This illustrates a tooth without substantial deterioration over time. The updated biomarker information consistently supported a low risk of tooth loss.

## 5 Discussion

Our proposed joint model was developed specifically for clustered data with one continuous biomarker, one binary biomarker, and a terminal event, motivated by tooth‐level clinical data in patients with periodontitis. We extended the joint modeling framework to make dynamic prediction for clustered survival outcomes with longitudinal biomarkers. We developed cluster‐informed dynamic prediction that incorporates longitudinal information from all units within a cluster to update individualized risk over time. Additionally, we evaluated predictive performance using time‐varying AUC and PE, appropriately accounting for censoring and clustering. This work contributes to the joint modeling and dynamic prediction literature by addressing multilevel correlation structure in both longitudinal and survival data.

In the simulation study, the proposed joint model substantially improved coverage probabilities compared to joint models implemented in JMbayes2 and rstanarm packages. We acknowledge that this comparison inherently favors the proposed method, as we generated data under the multilevel structure. Currently, JMbayes2 and rstanarm do not accommodate multilevel data structure and the implemented models are therefore misspecified. As a result, these packages assume independence of units in the survival submodel and produced estimates with narrower 95% credible intervals. Therefore, the model comparison should be viewed as an illustration of the impact of ignoring multilevel dependence in joint modeling. This highlights the importance of properly accounting for clustering when it is present in the data.

The predictive performance of the simulation study further support the proposed approach. The cluster‐informed dynamic prediction based on the multilevel joint model consistently achieved higher AUCs and lower PEs across multiple prediction windows. Moreover, as the frailty variance increased, the differences in predictive performance between the models became more pronounced, highlighting the advantage of incorporating cluster‐level information when within‐cluster dependence is stronger.

In the data application, most fixed‐effect estimates were similar between the proposed multilevel joint model and the JMbayes2 model. However, consistent with the simulation findings, the estimated standard deviations of many parameters were smaller under models that assume independence, suggesting that the model fails to adequately capture within‐cluster correlation and may underestimate uncertainty. This reinforces the importance of accounting for multilevel structure in practical applications involving clustered longitudinal and survival data.

It is worth noting that the random effects adopted in our joint model inherently assumed an exchangeable correlation structure among teeth within a patient, conditional on the molar and mandibular indicators. However, the progression of periodontitis may have a spatial pattern [39, 40], in which both the number and the location of missing teeth can be informative. Furthermore, individuals with advanced periodontitis may already have fewer teeth compared to individuals with less severe disease, introducing the presence of informative cluster size (ICS). A diseased tooth with high PPD or mobility can influence the health condition of a set of neighboring teeth instead of the entire mouth. Several methods have been developed to address ICS in clustered data by assigning weights to each cluster [16, 41, 42]. Reich et al. [43] developed a spatial model to account for the spatial dependence of continuous tooth‐level outcome. However, these methods have not been fully integrated into joint modeling frameworks that account for both multiple biomarkers and clustered survival outcomes. Reich and Bandyopadhyay [40] modeled periodontal biomarkers and missing tooth locations simultaneously using a shared latent spatial factor. However, their approach only considered PPD measurement at a single time point and did not account for time to tooth loss. Incorporating spatial dependence among units and accounting for ICS are needed in future development of joint modeling for clustered data with application to periodontitis. Another possible extension is to account for informative visit process as people with more severe disease may visit clinics more frequently. Lu et al. [19] developed a trivariate joint model for clustered data with longitudinal counts, recurrent events and a terminal event. Our future work may include a proportional hazard model for the gap time between visits, linked through shared random effects, to more accurately capture the dependence between visit frequency, biomarker trajectories, and the risk of tooth loss.

Our proposed joint model assumed linear trajectories for the biomarker, which may not fully capture the complex temporal patterns. In future work, we plan to incorporate spline functions to more flexibly capture longitudinal trajectories and to model time‐varying association structures between the longitudinal and survival processes [18, 44]. Computational burden remains a challenge due to the high dimension of random effects from multilevel data structure. A growing body of work has explored the use of integrated nested Laplace approximation (INLA) for fitting joint models [45, 46, 47, 48], which is computationally more efficient than Stan and MCMC approaches [49]. The INLA approach has primarily focused on efficient model estimation, but has not yet been extended to dynamic prediction. Extending the INLA framework to support dynamic prediction is another promising direction for future research.

In conclusion, the proposed joint model is well suited for studies in which multiple biomarkers evolve over time and affect clustered survival outcomes, as applied in periodontal research. The cluster‐informed dynamic prediction framework provides a valuable tool for improving predictive accuracy in clustered data settings. Overall, this work extends joint modeling methodology to multilevel structures and offers a practical approach for individualized risk prediction in multilevel clinical data.

## Funding

This work was supported by the Canadian Institutes of Health Research—Artificial Intelligence for Public Health (AI4PH) Training Platform and the Natural Sciences and Engineering Research Council of Canada—Discovery Grants Program (Grant No. RGPIN‐2022‐05356).

## Ethics Statement

The study was approved by the University of Toronto Research Ethics Board (RIS Human Protocol Number 43100).

## Conflicts of Interest

The authors declare no conflicts of interest.

## Supporting information




**Data S1:** Additional equations, figures, and tables referenced in Sections 2 to 4 are provided in the Supporting Information. **Figure S1:** Boxplots of IPCW time‐varying overall AUC and PE across different prediction windows, with the true standard deviation of frailty term assumed to be 1.2. The bold middle line represents the median. The lower and upper hinges correspond to the 25th and 75th percentiles. CI‐MLJM denotes the cluster‐informed prediction from the proposed multilevel joint model. UI‐MLJM denotes the unit‐informed prediction from the proposed multilevel joint model. JMbayes2 refers to unit‐informed prediction from JMbayes2 package, which assumes independence between units within each cluster. **Figure S2:** Boxplots of IPCW time‐varying overall AUC and PE across different prediction windows, with the true standard deviation of frailty term assumed to be 0.6. The bold middle line represents the median. The lower and upper hinges correspond to the 25th and 75th percentiles. CI‐MLJM denotes the cluster‐informed prediction from the proposed multilevel joint model. UI‐MLJM denotes the unit‐informed prediction from the proposed multilevel joint model. JMbayes2 refers to unit‐informed prediction from JMbayes2 package, which assumes independence between units within each cluster. **Figure S3:** Boxplots of IPCW time‐varying overall AUC and PE across different prediction windows, with the true standard deviation of frailty term assumed to be 0.2. The bold middle line represents the median. The lower and upper hinges correspond to the 25th and 75th percentiles. CI‐MLJM denotes the cluster‐informed prediction from the proposed multilevel joint model. UI‐MLJM denotes the unit‐informed prediction from the proposed multilevel joint model. JMbayes2 refers to unit‐informed prediction from JMbayes2 package, which assumes independence between units within each cluster. **Figure S4:** Time‐varying Receiver Operating Characteristic (ROC) curves for the prediction windows of year (4, 4.5], year (4, 5], year (4, 5.5], and year (4, 6] from the analysis of CAF data. CI‐Multilevel JM denotes the cluster‐informed prediction from the proposed multilevel joint model. JMbayes2 refers to the unit‐informed prediction from JMbayes2 package, which assumes independence between units within each cluster. **Table S1:** Results from the simulation study of model fitting. Multilevel JM denotes the proposed joint model for multilevel data. JMbayes2 refers to a joint model from JMbayes2 package, assuming independence between units within each cluster. Stan JM denotes a joint model from rstanarm package, assuming independence between units within each cluster. M‐Est is the mean of parameter estimates; Rel‐Bias is the ratio of average bias to the true value; M‐SD is the mean of the posterior standard deviation; and Coverage is the empirical coverage probability as the proportion of times the 95% credible interval contains the true value.

## Data Availability

The data that support the findings of this study are available on request from the corresponding author. The data are not publicly available due to privacy or ethical restrictions. Under agreement between the University of Toronto and the Canadian Forces Health Services, restrictions apply to the distribution and use of dental data from Canadian Armed Forces members that support the illustration of our methods. Simulated data along with R code to apply our proposed method are available at https://github.com/SeanFeng2333/Clustered‐JM‐Dynamic‐Prediction.git.
