# Supplementary material for: Joint Models of Two Longitudinal Biomarkers and Clustered Survival Data for Cluster‐Informed Dynamic Prediction With Application to Periodontitis
Source: Stat Med. 2026 Sep 8;45(20-22):e70715. doi: 10.1002/sim.70715 (PMC13551296; doi:10.1002/sim.70715)
Supplement: Supplementary file 1 [file SIM-45-0-s001.pdf]

Supporting Material for “Joint Models of Two Longitudinal Biomarkers and Clustered Survival Data for Cluster-Informed Dynamic Prediction with Application to Periodontitis”

Sean Xinyang Feng[1], Laurent Briollais[1,2], Aya A. Mitani[1]

[1] Division of Biostatistics, Dalla Lana School of Public Health, University of Toronto, Toronto, Canada

[2] Lunenfeld-Tanenbaum Research Institute, Mount Sinai Hospital, Toronto, Canada

Correspondence: Sean Xinyang Feng, xinyang.feng@mail.utoronto.ca

A Marginal Covariance Between the Linear Predictors

Given the above parametrization, the marginal covariance between the linear predictors, $\eta_{Aij}(t)$ and $\eta_{Bij}(t)$ are given by:

$$\mathrm{Cov}(\eta_{Aij}(t), \eta_{Aij}(t')) = \sigma^2_{CA} + \sigma^2_{UA0} + (t + t')\rho_1\sigma_{UA0}\sigma_{UA1} + tt'\sigma^2_{UA1}$$

$$\mathrm{Cov}(\eta_{Aij}(t), \eta_{Aij'}(t)) = \mathrm{Cov}(\eta_{Aij}(t), \eta_{Aij'}(t')) = \sigma^2_{CA}$$

$$\mathrm{Cov}(\eta_{Bij}(t), \eta_{Bij'}(t)) = \mathrm{Cov}(\eta_{Bij}(t), \eta_{Bij'}(t')) = \sigma^2_{CB}$$

$$\mathrm{Cov}(\eta_{Bij}(t), \eta_{Bij}(t')) = \sigma^2_{CB} + \sigma^2_{UB0} + (t + t')\rho_6\sigma_{UB0}\sigma_{UB1} + tt'\sigma^2_{UB1}$$

$$\mathrm{Cov}(\eta_{Aij}(t), \eta_{Bij}(t)) = \rho_C\sigma_{CA}\sigma_{CB} + \rho_2\sigma_{UA0}\sigma_{UB0} + t\rho_3\sigma_{UA0}\sigma_{UB1} + t\rho_4\sigma_{UA1}\sigma_{UB0} + t^2\rho_5\sigma_{UA1}\sigma_{UB1}$$

$$\mathrm{Cov}(\eta_{Aij}(t), \eta_{Bij}(t')) = \rho_C\sigma_{CA}\sigma_{CB} + \rho_2\sigma_{UA0}\sigma_{UB0} + t'\rho_3\sigma_{UA0}\sigma_{UB1} + t\rho_4\sigma_{UA1}\sigma_{UB0} + tt'\rho_5\sigma_{UA1}\sigma_{UB1}$$

$$\mathrm{Cov}(\eta_{Aij}(t), \eta_{Bij'}(t)) = \mathrm{Cov}(\eta_{Aij}(t), \eta_{Bij'}(t')) = \rho_C\sigma_{CA}\sigma_{CB}$$

B Dynamic Prediction Derivation

Details of obtaining Equation 8:

$$\begin{aligned}
\pi_{ij^*}(u|t) &= \Pr\{T^*_{ij^*} \geq u | T^*_{ij^*} > t, \boldsymbol{Y}_{ij^*}(t), \mathcal{D}_{i-j^*}(t), \mathcal{D}\} \\
&= \int \Pr\{T^*_{ij^*} \geq u | T^*_{ij^*} > t, \boldsymbol{Y}_{ij^*}(t), \mathcal{D}_{i-j^*}(t), \mathcal{D}; \boldsymbol{\theta}\} p(\boldsymbol{\theta}|\mathcal{D}) d\boldsymbol{\theta} \\
&= \int \Big[\int \Pr\{T^*_{ij^*} \geq u | T^*_{ij^*} > t, \boldsymbol{Y}_{ij^*}(t), \mathcal{D}_{i-j^*}(t), \boldsymbol{B}_i; \boldsymbol{\theta}\} p\{\boldsymbol{B}_i | T^*_{ij^*} > t, \boldsymbol{Y}_{ij^*}(t), \mathcal{D}_{i-j^*}(t); \boldsymbol{\theta}\} d\boldsymbol{B}_i \Big] p(\boldsymbol{\theta}|\mathcal{D}) d\boldsymbol{\theta} \\
&= \int \int \Pr(T^*_{ij^*} \geq u | T^*_{ij^*} > t, \boldsymbol{B}_i; \boldsymbol{\theta}) p\{\boldsymbol{B}_i | T^*_{ij^*} > t, \boldsymbol{Y}_{ij^*}(t), \mathcal{D}_{i-j^*}(t); \boldsymbol{\theta}\} p(\boldsymbol{\theta}|\mathcal{D}) d\boldsymbol{B}_i d\boldsymbol{\theta} \\
&= \int \frac{S_{ij^*}\{u | \mathcal{H}_{ij^*}(u, \boldsymbol{b}_i, \boldsymbol{b}_{ij^*}, r_i, \boldsymbol{\theta}); \boldsymbol{\theta}\}}{S_{ij^*}\{t | \mathcal{H}_{ij^*}(t, \boldsymbol{b}_i, \boldsymbol{b}_{ij^*}, r_i, \boldsymbol{\theta}); \boldsymbol{\theta}\}} p\{\boldsymbol{B}_i | T^*_{ij^*} > t, \boldsymbol{Y}_{ij^*}(t), \mathcal{D}_{i-j^*}(t); \boldsymbol{\theta}\} p(\boldsymbol{\theta}|\mathcal{D}) d(\boldsymbol{B}_i, \boldsymbol{\theta})
\end{aligned}$$

C Additional Simulation Results

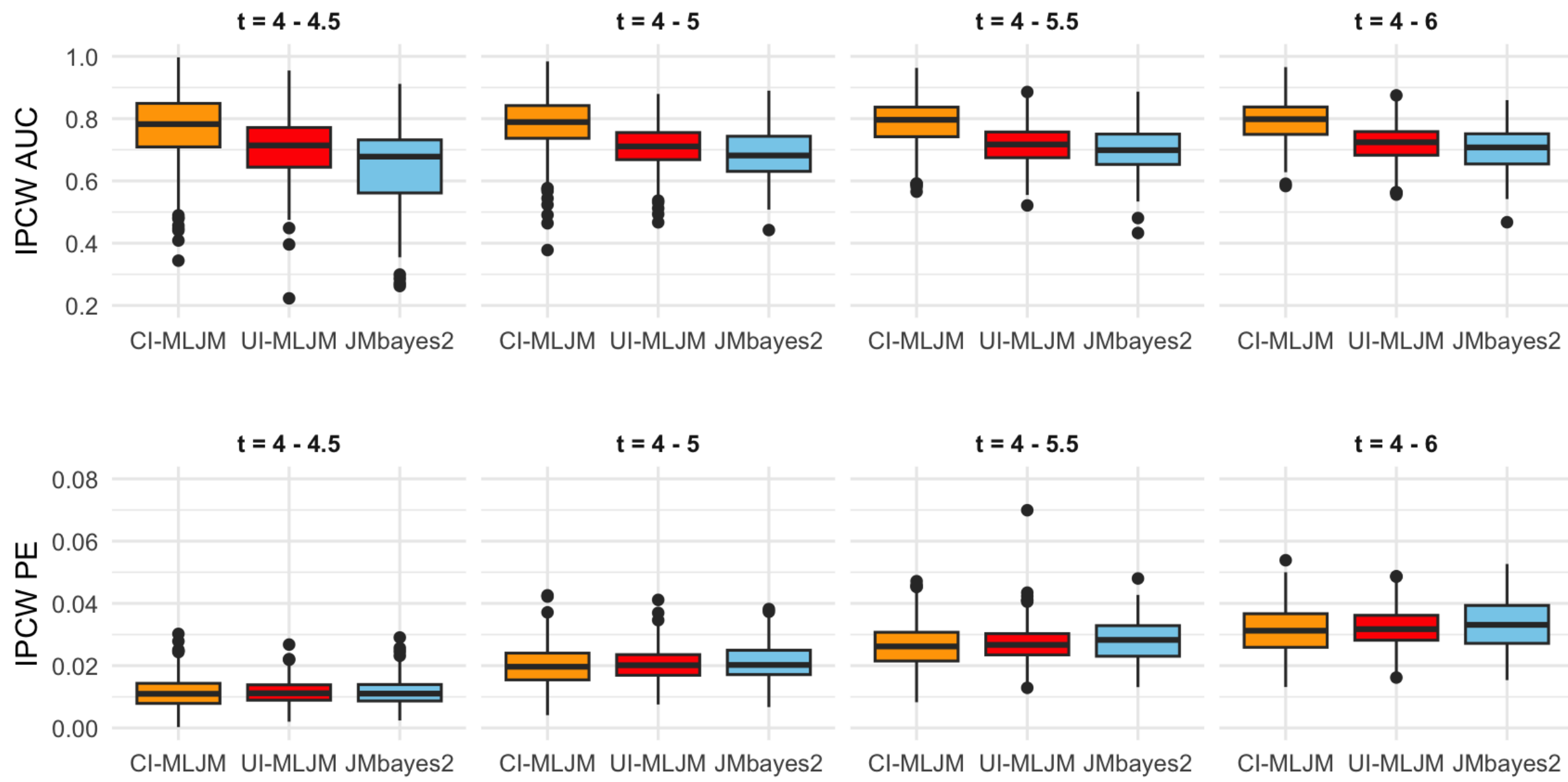

Figure S1: Boxplots of IPCW time-varying overall AUC and PE across different prediction windows, with the true standard deviation of frailty term assumed to be 1.2. The bold middle line represents the median. The lower and upper hinges correspond to the 25th and 75th percentiles. CI-MLJM denotes the cluster-informed prediction from the proposed multilevel joint model. UI-MLJM denotes the unit-informed prediction from the proposed multilevel joint model. JMbayes2 refers to unit-informed prediction from `JMbayes2` package, which assumes independence between units within each cluster.

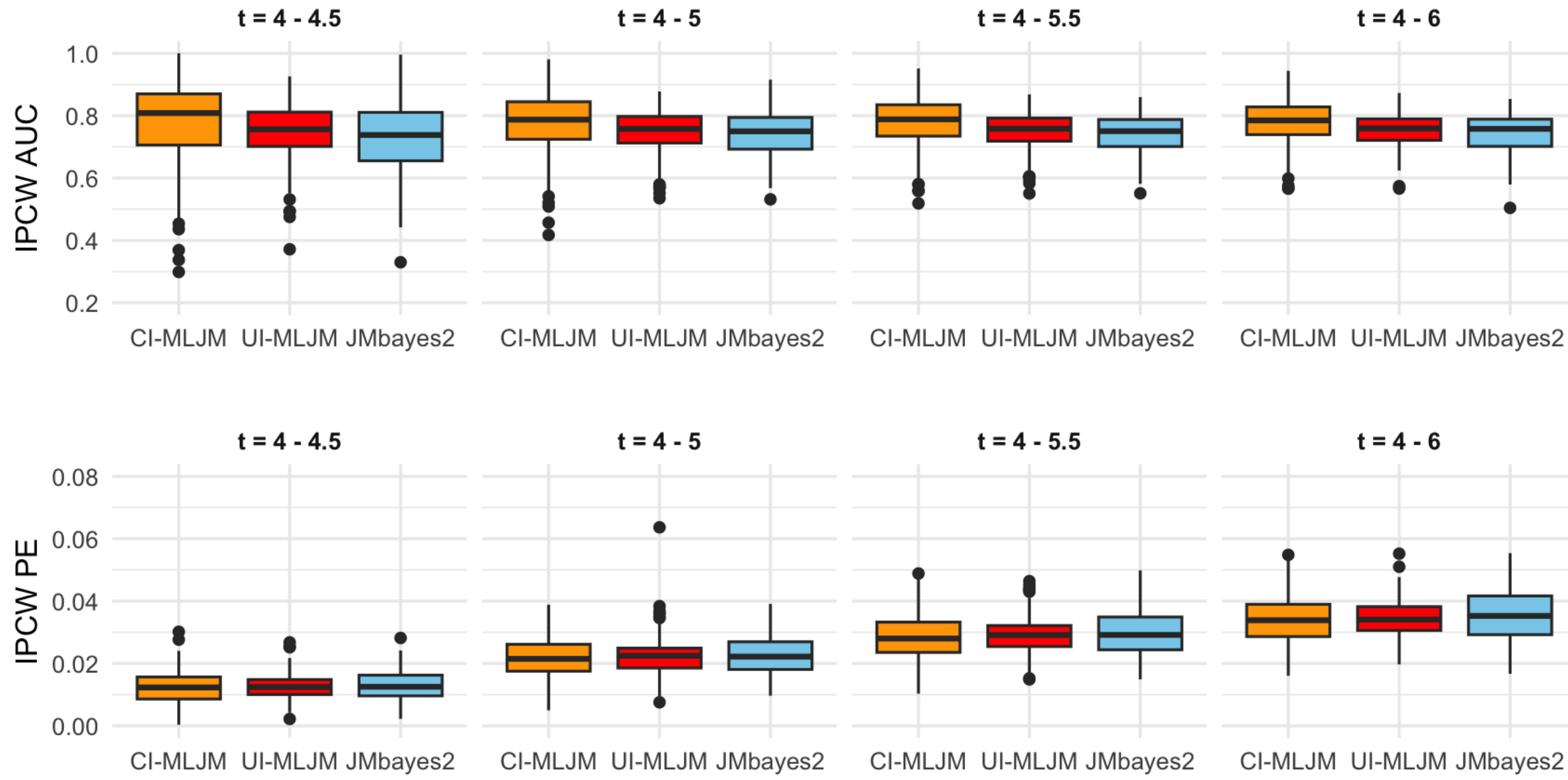

Figure S2: Boxplots of IPCW time-varying overall AUC and PE across different prediction windows, with the true standard deviation of frailty term assumed to be 0.6. The bold middle line represents the median. The lower and upper hinges correspond to the 25th and 75th percentiles. CI-MLJM denotes the cluster-informed prediction from the proposed multilevel joint model. UI-MLJM denotes the unit-informed prediction from the proposed multilevel joint model. JMbayes2 refers to unit-informed prediction from `JMbayes2` package, which assumes independence between units within each cluster.

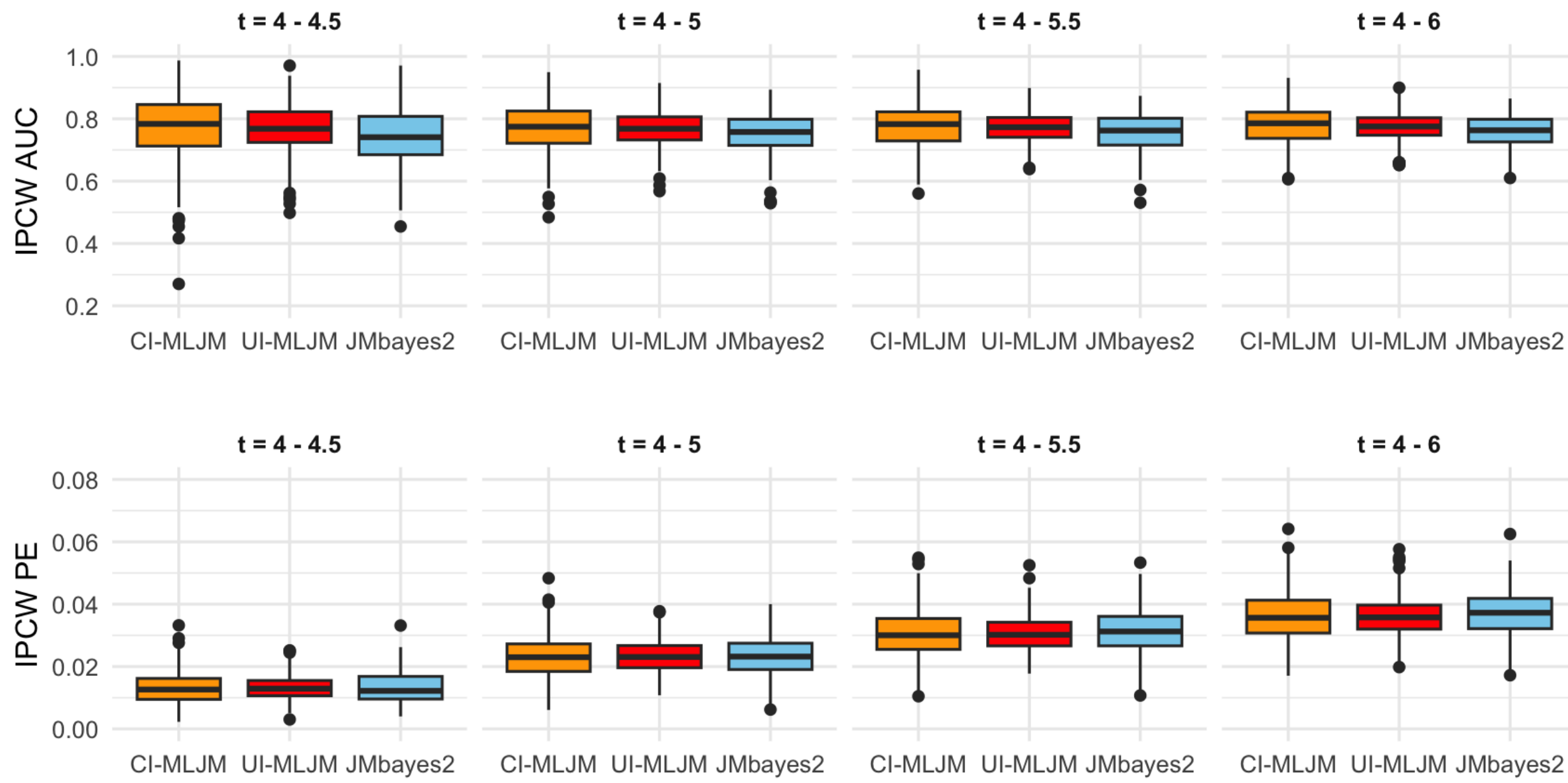

Figure S3: Boxplots of IPCW time-varying overall AUC and PE across different prediction windows, with the true standard deviation of frailty term assumed to be 0.2. The bold middle line represents the median. The lower and upper hinges correspond to the 25th and 75th percentiles. CI-MLJM denotes the cluster-informed prediction from the proposed multilevel joint model. UI-MLJM denotes the unit-informed prediction from the proposed multilevel joint model. JMbayes2 refers to unit-informed prediction from `JMbayes2` package, which assumes independence between units within each cluster.

		Multilevel JM				JMbayes2				Stan JM			
Parameter	True	M-Est	Rel-Bias	M-SD	Coverage	M-Est	Rel-Bias	M-SD	Coverage	M-Est	Rel-Bias	M-SD	Coverage
Survival submodel													
γ_0	-6.00	-6.084	0.014	0.203	0.912					-5.612	-0.065	0.262	0.592
γ_1	0.10	0.087	-0.129	0.075	0.954	0.085	-0.150	0.040	0.750	0.054	-0.459	0.039	0.643
γ_2	1.70	1.675	-0.015	0.092	0.951	1.527	-0.102	0.083	0.460	1.363	-0.198	0.121	0.122
α_1	4.00	4.068	0.017	0.154	0.922	3.656	-0.086	0.182	0.508	3.722	-0.070	0.216	0.602
α_2	0.13	0.130	-0.002	0.012	0.944	0.121	-0.068	0.008	0.751	0.110	-0.153	0.008	0.367
Longitudinal submodel (continuous biomarker)													
β_{AX0}	1.30	1.295	-0.004	0.024	0.952	1.300	0.000	0.007	0.600	1.297	-0.002	0.011	0.643
β_{AX1}	0.10	0.102	0.016	0.018	0.945	0.100	0.002	0.005	0.480	0.102	0.017	0.005	0.490
β_{AX2}	-0.10	-0.100	0.003	0.002	0.959	-0.100	0.000	0.002	0.938	-0.101	0.014	0.002	0.847
β_{AZ1}	0.30	0.300	-0.001	0.016	0.945	0.302	0.007	0.013	0.963	0.300	0.002	0.013	0.980
σ_e	0.20	0.204	0.020	0.008	0.959	0.200	0.000	0.001	0.971	0.203	0.013	0.005	1.000
Longitudinal submodel (binary biomarker)													
β_{BX0}	-0.80	-0.757	-0.054	0.438	0.956	-0.773	-0.034	0.203	0.621	-0.784	-0.020	0.205	0.653
β_{BX1}	-1.00	-0.966	-0.034	0.433	0.956	-0.999	-0.001	0.172	0.561	-0.977	-0.023	0.170	0.520
β_{BX2}	0.20	0.198	-0.011	0.039	0.952	0.203	0.015	0.039	0.947	0.166	-0.168	0.037	0.806
β_{BZ1}	-1.40	-1.381	-0.014	0.354	0.961	-1.340	-0.043	0.397	0.974	-1.402	0.001	0.392	0.990
Random effects													
σ_r	0.60	0.611	0.018	0.059	0.954								
σ_{CA}	0.15	0.153	0.023	0.018	0.944								
σ_{CB}	4.00	4.037	0.009	0.371	0.936								
ρ_C	0.50	0.472	-0.055	0.089	0.952								
σ_{UA0}	0.20	0.203	0.013	0.015	0.945	0.249	0.245	0.006	0.004	0.239	0.193	0.019	0.214
σ_{UA1}	0.03	0.036	0.211	0.012	0.956	0.030	0.004	0.002	0.987	0.031	0.022	0.003	0.959
σ_{UB0}	6.00	6.029	0.005	0.289	0.940	7.169	0.195	0.353	0.082	7.285	0.214	0.373	0.051
σ_{UB1}	0.60	0.609	0.015	0.040	0.945	0.603	0.005	0.036	0.913	0.617	0.028	0.034	0.918
ρ_1	-0.50	-0.490	-0.020	0.042	0.938	-0.397	-0.206	0.043	0.384	-0.405	-0.189	0.068	0.439
ρ_2	0.20	0.192	-0.039	0.043	0.947	0.298	0.488	0.026	0.133	-0.002	-1.009	0.000	0.000
ρ_3	0.10	0.104	0.045	0.069	0.933	0.102	0.017	0.073	0.943	-0.021	-1.206	0.004	0.000
ρ_4	0.10	0.106	0.061	0.060	0.944	0.081	-0.190	0.053	0.892	-0.013	-1.134	0.003	0.000
ρ_5	0.30	0.300	-0.001	0.072	0.956	0.307	0.025	0.066	0.981	-0.158	-1.528	0.033	0.010
ρ_6	-0.40	-0.379	-0.053	0.070	0.949	-0.275	-0.313	0.105	0.822	-0.001	-0.998	0.000	0.000

Table S1: Results from the simulation study of model fitting. Multilevel JM denotes the proposed joint model for multilevel data. JMbayes2 refers to a joint model from `JMbayes2` package, assuming independence between units within each cluster. Stan JM denotes a joint model from `rstanarm` package, assuming independence between units within each cluster. M-Est is the mean of parameter estimates; Rel-Bias is the ratio of average bias to the true value; M-SD is the mean of the posterior standard deviation; and Coverage is the empirical coverage probability as the proportion of times the 95% credible interval contains the true value.

D Additional Data Application Results

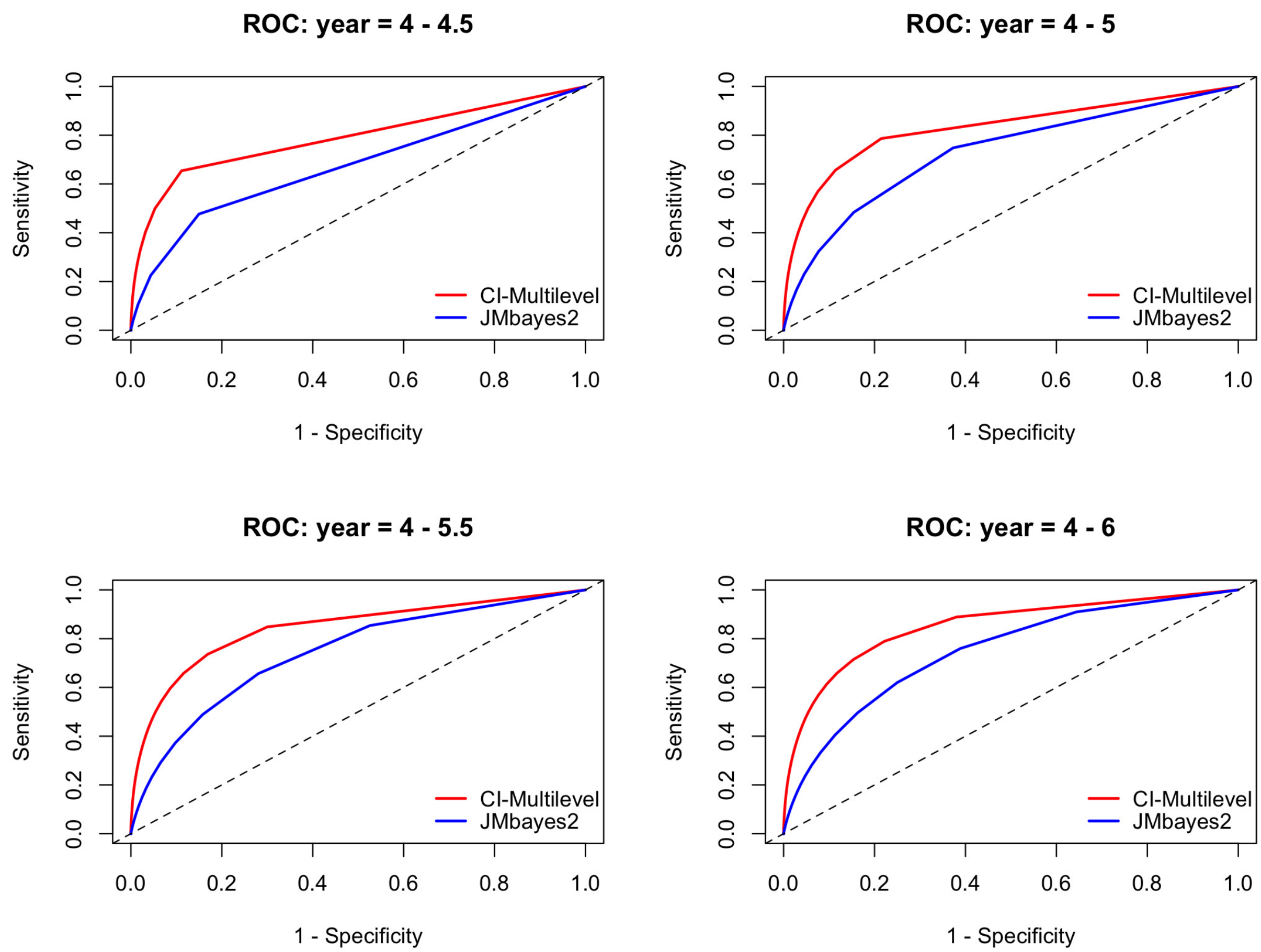

Figure S4: Time-varying Receiver Operating Characteristic (ROC) curves for the prediction windows of year (4, 4.5], year (4, 5], year (4, 5.5], and year (4, 6] from the analysis of CAF data. CI-Multilevel JM denotes the cluster-informed prediction from the proposed multilevel joint model. JMbayes2 refers to the unit-informed prediction from `JMbayes2` package, which assumes independence between units within each cluster.
